# Prevalence and correlates of depression among Australian women: a systematic literature review, January 1999- January 2010

**DOI:** 10.1186/1756-0500-6-424

**Published:** 2013-10-21

**Authors:** Jane L Rich, Jennifer M Byrne, Cassie Curryer, Julie E Byles, Deborah Loxton

**Affiliations:** 1 Research Centre for Gender, Health & Ageing, Faculty of Health and Medicine, University of Newcastle, Callaghan, NSW 2308, Australia; 2 Hunter Medical Research Institute Public Health Capacity Building Group, University of Newcastle, Callaghan, NSW 2308, Australia; 3 Australian Longitudinal Study on Women’s Health, The University of Newcastle, Callaghan, NSW 2308, Australia

**Keywords:** Gender, Mental health, Age-span, Abuse, Diverse

## Abstract

**Background:**

Little is known about the prevalence and correlates of depression among Australian women. This systematic review of depression among women in Australia, the largest identified to date, highlights the prevalence and correlates of depression across the life span.

**Results:**

The report adhered to the Preferred Reporting Items for Systematic Reviews and Meta-Analyses: The PRISMA Statement (PRISMA). Six health related databases were selected: Medline, PsychInfo, SCOPUS, Cinhal, Informit and Cochrane Systematic Reviews. 1,888 initial articles were found, and 111 articles were considered relevant for review. Prevalence rates of depression among women ranged from 2.6% to 43.9%. Higher rates were reported for younger women, or specific population groups. Most significant correlates included, age, adverse life events, tobacco use, sole motherhood, and previous mental health problems.

**Conclusions:**

Limitations include the scope of the investigation’s aims and inclusion criteria, and the failure to identify gender specific data in most studies. Publication bias was likely, given that only papers reported (or translated) in English were included. Despite the breadth of information available, there were noticeable gaps in the literature. Some studies reported on affective disorders, but did not specifically report on depression; it is concluded that each mental illness warrants separate investigation. It was also common for studies to report a total prevalence rate without separating gender. This report recommends that it is vital to separate male and female data. The report concludes that more research is needed among mid-age women, Indigenous women, non-heterosexual women and Culturally and Linguistically Diverse (CALD) women.

## Background

Around one million adults in Australia live with depression each year [1]. Depression has adverse effects on both mental and physical health [2], and is thought to contribute to and increase the risk of comorbid chronic health conditions such as cardiovascular disease and Type 2 Diabetes which in turn contributes to the onset of depression [3-5]. Epidemiological and clinical studies have shown that women experience depression at significantly greater numbers than men [6]; with varying prevalence rates. Women are particularly vulnerable to experiencing depression in the time surrounding the birth of a child [7]. Postnatal depression affects almost 16 per cent of new mothers [7]. Moreover, the impact of gender roles, family, and caring responsibilities can impact on women’s coping capacities and the demands of depression-related illness [8]. It is important therefore, to increase knowledge about depression among women, and to enhance health service responsiveness towards the needs of women living with, or at risk of developing, depression. This report will present the prevalence and correlates of depression for women in Australia.

A considerable body of research has already been undertaken on the prevalence of depression and associated risk factors. However, variations in research methodology and measurement instruments have resulted in different prevalence estimates and associations. For example, prevalence rates for depression among women have been reported as ranging from 4.3% of the population [9] to as high as 43.9% [10]. There is a lack of systematic summary of the range of prevalence estimates from different studies. Some recent reviews have been undertaken in specific population groups. For example, Godart et al.'s [1] systematic review assessed the prevalence of mood disorders in females with an eating disorder, and Luppa et al.'s [2], systematic review of age and gender specific rates of depression among persons aged 75 and over. However, these reviews do not provide an overview of depression among women in the general population, that can more accurately inform policy, program and research initiatives.

### Aims of the systematic review

The aims of the current systematic review were to gauge the prevalence and correlates of depression among Australian women over 12 years of age. The current review particularly focused on different age ranges, women from Indigenous and culturally and linguistically diverse backgrounds, and women living in rural areas. In addition, the current review aimed to:

address the lack of recent large-scale systematic literature reviews on the prevalence and correlates of depression among Australian women over 12 years of age;

highlight those areas of knowledge which are currently under-researched, and thus inform the development of targeted research strategies; and

communicate to a wider audience evidence-based information about the prevalence and correlates of depression among a diverse population of women in Australia, to inform health services, policy and professional practice.

This paper draws upon an earlier report that was prepared for *beyondblue*, an independent, non-profit organisation in Australia dedicated to education and advocacy for people and their carers who are living with depression, anxiety and related substance misuse; the development of prevention, early intervention and research programs; and support and training for GPs and other allied health professionals. Further information about *beyondblue* is available at http://www.beyondblue.org.au

### Measurement of depression

The gold standard of depression diagnosis is The American Psychiatric Association’s *Diagnostic and Statistical Manual of Mental Disorders*[11,12], and the World Health Organisation’s *International Statistical Classification of Diseases and Related Health Problems*[13,14]. The *Diagnostic and Statistical Manual of Mental Disorders* describes experiences of depression as low moods, diminished interest or pleasure in activities, insomnia, fatigue, feelings of worthlessness, thoughts of death and diminished ability to concentrate [11].

Common measures of depression for research purposes include scales and checklists that are administered via self-report and interview techniques, those featured in this review are listed in Additional file 1: Table S1. Scales and checklists usually describe various depressive symptoms, and the respondent is asked to indicate how often or to what extent they have felt a particular way, within a specific time period. Higher numbers of symptoms and high frequency of symptoms generally indicates a greater degree of depressive symptoms and higher likelihood of a clinical level of depression.

Although the scope of the current review does not allow an in-depth comparison of depression measures, it is important to note that different measurement techniques will give rise to different prevalence estimates. For example, the mode of administration (self-report versus interview) and the type of measurement instruments used can lead to similar but not identical findings. Moreover, even when the same measurement instruments and methods are employed, scoring of data may differ. For example, many depression scales have cut-points that indicate the probable presence of depression; and even when the samples are drawn from the same or similar populations, different cut-points in different studies will inevitably result in different prevalence rates. In the current review, the measurement instruments used to determine the degree of depressive symptoms and the probable presence of depression are detailed in each relevant section.

## Methods

**Figure 1:**
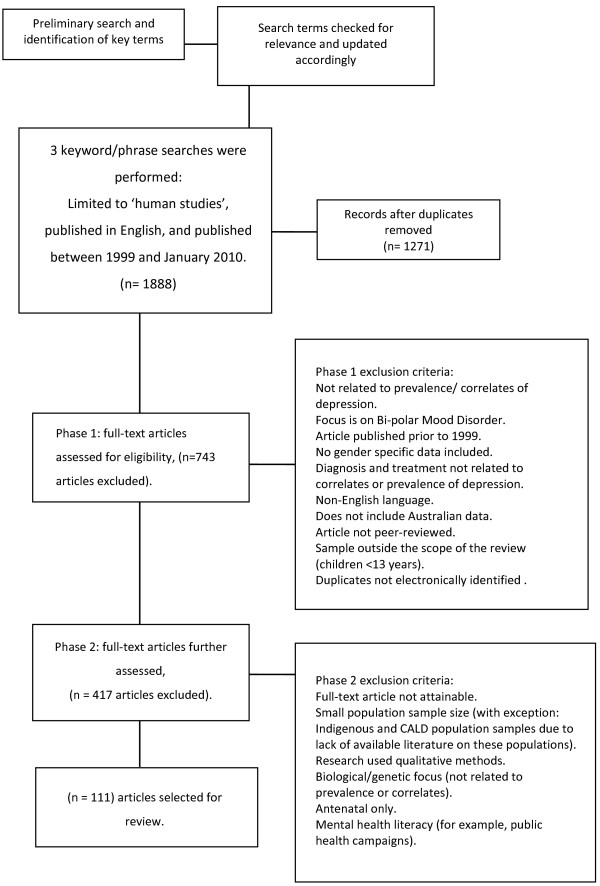
PRISMA flow chart.

This report was completed in accordance with the systematic review guidelines from the Preferred Reporting Items for Systematic Reviews and Meta-Analyses (PRISMA) group Figure 1[15].

### Search parameters

Prior to the search, Journals, Reference Databases, websites and key authors were identified by the project team, with six health-related databases selected for searching. Simple electronic database searches were undertaken of Medline, Psychinfo, SCOPUS, Cinhal, Informit (Health and Indigenous) and Cochrane Systematic Reviews to obtain pertinent keywords, and mesh subject headings where applicable. Wildcards (denoted as *) and keywords were searched for in title, original title, abstract, name of substance word and subject heading, used separately to ensure relevance, and then in combination with the word ‘or’. More information on the preliminary search terms used is shown in Additional file 1: Table S2.

The relevance of these terms were ascertained, and search terms updated accordingly. Three further electronic searches were then performed of the databases Medline, Psychinfo, SCOPUS, Cinhal, Informit (Health and Indigenous) and Cochrane Systematic Review., using the following terms: ‘depress*’ and ‘(wom*n* or female* or girl*)’ and ‘Australia*’ and ‘(prevalence or correlat*)’; ‘depress*’ and ‘(wom*n* or female* or girl*)’ and ‘Australia*’ and ‘aborig*’ or ‘Indigenous’ and ‘therap*’; and ‘wom*n’ or ‘female*’ or ‘girl*’AND ‘Therap*’ OR ‘diagnosis’ OR ‘treatment’ OR ‘psychotherap*’ AND ‘depress*’ AND ‘Australia*’. All searches were limited to ‘human’ studies, and were published in English, between 1999 and January 2010 (see Figure 1).

### Inclusion and exclusion criteria

Protocols for the selection of articles applied inclusion and exclusion criteria developed and agreed to by the project team, following review of a random sample of readings. Inclusion criteria were those articles focused on prevalence and correlates of depression, articles that included general population samples, data presented separately for males and females, articles that included data from Australia, peer reviewed, and available as full text articles (either electronically or hard copy). Exclusion criteria were: articles published prior to 1999, not available in English, only included children aged under 13 years or only included pregnant women, focus on bipolar mood disorder, articles about diagnosis, treatment and not about prevalence or correlates, articles about biological or genetic factors, articles where data were not presented by gender, articles that did not include Australian data, and duplicates that were not identified electronically. Studies with small sample sizes (less than 50 participants), or involving non-representative samples, and qualitative studies were not included in the general reviews unless they related to specific population groups that are otherwise underrepresented in population samples (for example, Indigenous women, women from non-English speaking backgrounds (NESB), prisoners). National reports were included, for example, the Australian Bureau of Statistics (ABS) and Australian Institute of Health and Welfare (AIHW) reports, as they were the main source of national data and were often published outside of academic journals.

### Categorisation

Selected articles were classified into seven pre-determined categories. These categories inform the organisational structure of the current review, namely: prevalence and correlates of depression; prevalence and correlates of depression at different ages and life stages including depression among young women; pregnancy-related depression; depression among middle-aged women; depression among older women; depression among Indigenous and culturally and linguistically diverse women; and rurality and depression. These categories are discussed further in the current review. As some articles covered multiple topic areas, some articles were placed in more than one category. This review has presented the information, correlates and prevalence rates of depression as found in the literature. The authors are aware that comparisons cannot be drawn between different studies and samples. This is why tables are presented below and in the Additional files 1 for each category that report the samples and measures used in each study.

## Results

A total of 1,888 citations were downloaded into a single library in Endnote [4]. After 617 duplicate citations were removed, 1,271 articles were screened using inclusion and exclusion criteria (see Additional file 1: Table S3). Of these, 111 articles were retained for inclusion in the main body of the review. Details of the included studies are summarised throughout the following subsections of analysis. Table 1 provides a summary of articles that explore the prevalence and correlates of depression generally, while the tables in the Additional file 1 explore articles specific to classified groups of women in Australia. Of the 111 articles included in this review, a small proportion of studies were longitudinal in nature [16-23]. Study samples ranged in size, the smallest being a study on 52 women [24], to as many as 21,900 participants [25]. Not all articles focused exclusively on women, for example, Kerse et al. and Tang et al., [25,26], and Pirkis et al's [27] study of depression among 20,226 community dwelling older people, of whom 12,880 were female.

**Table 1:** Prevalence and correlates of depression in Australian women

**Reference**	**Sample characteristics**	**Prevalence**	**Main findings**
Alati et al. [28]	Cross sectional survey of patients aged 16–84 years presenting for treatment over a 14 day period to a Gold Coast Hospital Emergency Department.		Using the Hospital Anxiety and Depression Scale (HADS) to measure state anxiety and depression revealed a linear relationship between alcohol consumption and anxiety and depression.
Alati et al. [16]	Mothers were recruited to the Mater University Study of Pregnancy (MUSP) at the Mater Misericordiae Hospital in Brisbane at their first antenatal visit and followed up at 3–5 days, 6 months, and 5, 14 and 21 years after the birth of their child. Babies numbered 7,223, of which 48% were females.		The Centre for Epidemiologic Studies Depression Scale (CES-D) was used with 3,843 participants, 53% of the original sample. At 21 year follow-up, multivariate analyses showed a strong graded inverse association with birth weight and depression for females, even when all other potential confounding factors were adjusted for (OR 0.82, CI 0.73- 0.92).
Australian Institute of Health and Welfare [29]	There were 3.5 million young people aged 12.24 years in Australia (approximately 18% of the total population as of June 2001).		Reports a relationship between weight, body image, and depression among young women.
Brown and Lumley [30]	1,366 women were mailed a questionnaire from maternity hospitals and home birth practitioners in Victoria. 225 women responded, 204 of which participated in a follow-up telephone inteview at 7–9 months postpartum.		Poorer levels of emotional wellbeing were associated with tiredness, urinary incontinence and more minor illnesses than usual.
Butterworth [31]	Analysis of data from the Australian National Survey of Mental Health and Wellbeing (1997). The representative sample comprised 2,232 women with children who had completed the Composite International Diagnostic Interview, and included 622 lone mothers, and 1,610 partnered mothers (aged over 18 years).	Approx 18% of lone mothers experienced depressive disorders compared with 8% partnered mothers. Depression was measured using the CIDI.	Lone mothers were more likely to have psychiatric disorders (OR= 2.4 - 3.4) and to have experienced physical and sexual violence (OR= 3.1- 4.1) than partnered mothers. The measures of physical and sexual violence were better predictors of psychiatric disorders than either lone parent status or the sociodemographic measures.
Cheok et al. [32]	1,455 cardiac patients (aged 18–84 years) admitted to the cardiology unit in one of four major public hospitals in South Australia between August 2000 and December 2001. Patients were sent follow-up questionnaires at 3, 6 and 12 months. The Mean age of the participants was 62.2 years, and 68% of participants were men.	46.3% of 1,455 participants were classified as depressed on the Center for Epidemiological Studies Depression Scale (CESD) Scale or the Hospital Anxiety and Depression (HAD) Scale, and 19.4% of participants had CESD scores that were suggestive of major depression. 54% of female participants were classified as depressed.	Elevated scores were associated with being younger, female, divorced or separated, not employed, living alone, having a lower level of education, and having poorer health and quality of life.
Gillespie et al. [33]	Two cohorts of women (N = 8,077, aged 18 – 45 years) from the Australian National Health and Medical Research Council Twin Register.		Age and depression were negatively correlated (r = −0.20), suggesting that symptoms of depression decrease over time.
Goldney et al. [34]	Random but representative sample of 3,010 South Australian residents aged 15 years and over. Participants were recruited by selecting 10 dwellings beginning with every fourth household from a random starting point in a number of metropolitan and rural districts.	Of the 3,010 male and female respondents, 204 (6.8%) had major depression (8.1% females). 132 (4.4%) had major depression alone (5.3% females); 146 (4.9%) had dysthymia (5.1% females); 62 (2.1%) had dysthymia alone (1.9% females); and 66 (2.2 %) had double depression (2.5% females). Depression was measured using the SF-36, the AQoL and the mood module of the PRIME-MD.	Overall, more females (8.1%) than males (5.4%) had major depression.
Goldney et al. [35]	Random and representative sample of 3,015 South Australians aged 15 years and over when recruited for Goldney et al’s. [35] survey.	There was no significant change in the prevalence of depression from 1998 to 2004. Depression was measured using the mood module of the PRIME-MD and the AoQL.	
Goldney et al. [36]	Random and representative sample of 2,501 South Australians (aged 18 years and over) randomly selected from the telephone directory. Suicidal ideation and clinical depression were determined by the general health questionnaire (GHQ-28) and the short-form health survey (SF-12).	Overall, 5.6% of men and 5.3% of women had experienced suicidal ideation. Depression was measured using the GHQ-28.	Depression was strongly associated with suicidal ideation. Traumatic events are a significant factor that contributes to suicidal ideation.
Hawthorne et al. [37]	1998 Health Omnibus Data. 3,010 interviews conducted with people aged over 15 years.	For females, the 12 month prevalence of major depression was 8% and 11% for ‘other’ depression. Depression was measured using the PRIME-MD.	
Hegarty et al. [38]	1,257 female patients (aged 16–50 years) attending general practitioners.	18% of women reported sufficient depressive symptoms to be considered ‘probably’ depressed. Depression was measured using the BDI or EPDS.	Depressed women were more likely to have experienced severe combined abuse than women who were not depressed.
Henderson et al. [39]	Household sample of 10,600 persons aged 18 or over were interviewed by the Australian Bureau of Statistics (ABS).	The 12 month prevalence of depression in women was 12%. The prevalence of depressive disorders was lower in persons aged 65 and over. Depression was measured using the CIDI-A.	Of women with a depressive disorder, 57% had at least one other mental health disorder.
Herrman et al. [40]	18,489 primary care patients aged 18 to 75 years, across six countries (Israel, Brazil, Australia, Spain, Russian Federation and USA).		Higher depression scores were consistently associated with poorer health, functional status, quality of life and increased health care use. When age, marital status, and education level were controlled for, having a score equal to or over 16 (the cut-point for depression) was more likely for women than men in all sites except Melbourne.
Jirojwong et al. [41]	A cross-sectional study of 143 women (Mean age 28 years) recruited from two regional hospitals in Queensland.		There was a positive correlation between the number of follow-up home visits and depressive symptoms among women who gave birth at one hospital but not the other hospital.
Khawaja and Duncanson [42].	287 university students in Queensland (M = 26.32 years), 221 (77%) female. Caucasian students numbered 205 (71%), 57 (20%) were Asian students, and 24 (8%) were from other cultures.		Females had a significantly higher mean level of depressive symptoms when compared with males.
Kirk et al. [43]	Community based sample of 2,703 Australian twins over the age of 50 years (female N = 1,873).		Factor analysis was used to consider the relationship between fatigue, anxiety, and depression. Results suggested that fatigue could be considered a separate syndrome that is correlated with depression and anxiety, rather than merely as a symptom of depression or anxiety.
LaMontagne et al. [44]. "Job strain - Attributable depression in a sample of working Australians: Assessing the contribution to health inequalities." BMC Public Health 8.	Telephone survey of working Victorians aged over 18 years (N = 1,101).		Job strain was found to contribute to depression.
Middeldorp et al. [45]	Data collected from the Australian and Netherlands Twin Registers (N = 2,470; female N = 1,402) and (N = 1,256; female N = 686) respectively.		In both countries, depressive disorders were more common among women than men.
Migliorini et al. [46]	443 community dwelling adults (28% female, Mean age 52 years) with a spinal cord injury recruited from a spinal cord injury registry. Results were compared to normative Depression, Anxiety and Stress Scale (DASS-21) data obtained from a large adult non-clinical United Kingdom (UK) population (N = 1794; male N = 815, Mean age 41.0 years).	The prevalence of depression for the total sample was 37% (including males). Depression was measured using the DASS-42.	Relative to the normative sample, females with spinal cord injuries were slightly more likely than males to experience depression. In comparison to the normative sample, the odds ratio (OR) for any Depression was OR 2.09 (95%CI, 1.67-2.62).
Tye and Mullen [47]	103 women (Mean age 29.6 years) from Victorian prisons in Australia were interviewed.	44% of the sample met the criteria for major depression. Depression was measured using the CIDI and the PDQ-4+.	Female prisoners had significantly higher rates of depression compared with women in the community.

### Prevalence and correlates of depression

The findings of the current systematic review show that where males and females were compared, generally, women are more likely to experience depression compared to men [10,34,35,48]. However, some studies did not concur, for example Kikkinen et al. [49] found no significant differences in prevalence rates between men and women living in rural areas, suggesting that rural males and females faced similar levels of risk (overall prevalence of 31%).

Among women, prevalence rates were found to range from 4.3% [9] to 43.9% [10]. Prevalence rates also varied by age, with older women overall appearing at less risk of experiencing depression (1.9% in the 60–64 year age group compared to 6% of 20–24 year olds and 4.4% of 40–44 year old women respectively) [9]. Higher rates of depression were reported from studies involving particular population groups such as Indigenous women [24,50] and those with chronic disease such as CVD [51]. It is important to note that different scales were used to measure prevalence rates, although the scales were appropriate for the target groups. It is unclear whether the reduced likelihood of depression in older age is attributable to cohort or age effects, thus further longitudinal research is indicated in this area. Different stages of life were also found to involve event or age-specific correlates of and risk factors for the onset of depression. For instance, depression was associated with skin problems [21] and poor body image [29] among younger women, surgical menopause among middle aged women [52], and dental problems [53] among older women.

For all age groups and stages of life, trauma and stressful life events were consistently associated with depression. In particular, strong associations exist between depression and violence and abuse, both as an adult and in childhood [31,38,39,48,54-57]. In addition [20] findings concluded that separation and divorce is associated with depression; as is sole motherhood [54,55]. However, being in an intimate relationship provides protection against depression [20].

At least some of the association between depression and sole motherhood is attributable to financial stress [54,55], which along with unemployment [24], has been significantly associated with depression [58,59]. Other demographic factors correlated with depression include educational and/or professional qualifications, or lack thereof [60], and place of birth [24,60-63].

The existence of previous depression [9] and other mental health illnesses including anxiety [39,64] are predictive of developing later depression. Chronic physical health problems, such as arthritis [65], angina [51], being a cardiac in-patient [32] and incontinence [30], have been found to be significantly correlated with depression. However, it is currently unclear whether depression precedes or is a consequence of chronic illness, and therefore, research into those conditions that are comorbid would benefit from comprehensive and longitudinal investigations to more fully understand these conditions. Current international literature does suggest this relationship is bi-directional [4,5,8].

The associations between age, stage of life, life events, demographics, health behaviour, chronic illness and depression suggest the existence of complex pathways that precede depression, and a variety of consequences that occur subsequent to depression, some of which may exacerbate and complicate the treatment of existing conditions. Thus, more comprehensive examinations of the pathways into, and the consequences of depression are indicated, to shed light on causal factors and to reveal possible mechanisms to optimise recovery from depression.

### Health behaviours

Health behaviours such as tobacco use, alcohol and drug use are well-known correlates of depression. For example, tobacco use is strongly and consistently associated with depression [66,67], as is alcohol use [17,59] and illicit drug use [59]. Whilst physical activity has been proven protective of depression [59,68], some studies indicate mixed results, with the protective effect most felt by women who are of healthy weight or overweight only, compared to women who are obese [69]. Nevertheless, even after adjusting for physical activity levels, being overweight and obesity are strongly associated with depression [70].

## Depression among women at different ages

### Depression among young women aged up to 32 years

This section of the current review details the literature on the prevalence and correlates of depression among a population of young women in Australia [29], identified as being between 12 and 32 years of age. The average age of the women in this section was calculated to be 20 years of age.

Previous research has found a higher prevalence of depression among young women, compared to those in middle- or older age [58,67]. In this review, estimates of depression among women aged 12 years and over range from 3.18% based on the Goldberg Depression Scale [6] to 30% using the CESD-10 [59]. Moreover, as young women reach the later stages of puberty their risk of experiencing depressive symptoms increases [71]. Young high school aged women experience a higher risk of depression than young men, with prevalence rates of 22% [72,73], whilst women in their twenties are also more likely to experience depressive symptoms than men in their twenties [10,42]. Young Asian Australian [74] and Indigenous Australian [24] women are particularly vulnerable to depression (53.8%) [24], having higher rates of prevalence than the general population, and these findings are discussed further in the specific topic sections in this review.

Two representative studies examined a range of correlates of depression. Among women aged 15 to 24 years, parental problems, sexual abuse, sexual identity conflict, financial difficulty, relationship break-downs, bullying, scholastic failure, and introversion were significantly associated with depression [75]. For those women aged 22 to 27 years, higher levels of depressive symptomatology were associated with lower socio-economic level, unemployment, low educational level, being single, having high health services use, illicit drug and alcohol use, and smoking [59].

For young women in this age group, particular life events for example unemployment [24], relationship issues, separation and divorce, abortion or miscarriage, and the onset of sole motherhood, have been associated with depression [20,54,55]. Bottomley et al's. [76] study found that 30% of pregnant adolescents aged between 13 and 20 years were at risk of depression during pregnancy. Moreover, for young women in this age group, past experience of abuse is related to both depression and suicidality [56].

For young women, issues surrounding weight and diet are of particular concern. Poor body image and perceptions of being overweight during adolescence are a significant risk factor for depression in young women [29,77], as is actually being overweight or obese [69], or conversely, being underweight [29,77]. Frequent dieting [78], and restrictive dietary practices, such as vegetarian diets [79] have also been found to be associated with depression. 21% of young women on vegetarian diets experience depression; compared with 15% of women non-vegetarians [79].

The absence of physical activity is also associated with the presence of depression [67], however, whilst physical activity has been found to be protective of depression [59,68], this effect may only apply to those women who are already at a healthy weight or alternatively, overweight (as opposed to obese) [69]. While much research into weight and diet issues already exists, detailed investigation into the factors that underlie body image and weight problems would advance the development of highly targeted, positive, and responsive intervention strategies. Young women are at particular risk of weight issues and poor body image, and by association, increased risk of depression, and therefore targeted intervention programs may help to prevent depression at a young age, and the subsequent development of depression at later ages or stages of the life course, such as following the birth of their first child, or as they transition into middle age.

Overall, the literature offered reasonable coverage of the prevalence and correlates of depression in this age group. However, some areas that have been linked with depression warrant further investigation, including skin problems [21], young pregnancy [76], and pregnancy losses [80]. Few studies have examined women’s transitions through life events [20], and therefore more longitudinal studies of this nature are warranted to determine those times when women are most vulnerable to depression. Additionally, an exploratory study with women in these age groups may help to identify those factors that the young women themselves implicate in the development of depression.

### Depression in the pregnancy-related period

Depression during the pregnancy-related stage is a serious illness that impacts adversely on women’s quality of life [81], on social and role functioning, and on the mother’s ability to care for and experience a positive maternal relationship with her child [81]. Following the birth of a child, around 70% of women will experience a short period of depressed mood commonly known as the ‘baby blues’. This depressed mood can last up to ten days, but is not considered to be postnatal depression or depression [12]. Postnatal depression occurs within four to six weeks following the birth of a child and includes all of the symptoms of major depression, and potentially, disinterest in or fearfulness of the baby [12]. Prevalence rates for postnatal depression among Australian women overall are 7.5% at six to eight weeks postpartum, however, point prevalence rates vary between states (from 10.2% in Queensland and South Australia to 5.6% in Western Australia) [82]. Among certain groups of women, the prevalence of pregnancy-related depression is higher than that found for the general population. For example, women from Indigenous and culturally and linguistically diverse backgrounds [83-85], and sole mothers experience significantly higher rates of postnatal depression (18%), compared to 8% of mothers with partners [31].

From a life course perspective, the transition into motherhood increases the likelihood of women experiencing depression [20]. Women who have previously experienced mental health problems [22] and those with a family history of mental health problems [86,87] are most likely to experience pregnancy-related depression, which in turn increases the risk of developing depression at later life stages [22].

Some research also suggests that lower socio-economic status, and for example, accessing public hospital as opposed to private hospital services [82] is associated with depression. However, living in an affluent area has also been found to be associated with depression [88], and so the relationship between depression and socio-economic status, health care affordability and hospital choice warrants further investigation. More research is also indicated to determine the role of demographic profiling in identifying those women most susceptible to postnatal depression.

Clear evidence of a link between infant temperament and postnatal depression has also been found. Hiscock and Wake’s [89] study found that 46% of mothers considered their infant’s sleep as problematic, with behaviours ranging from sleeping in the parent’s bed, having to be nursed to sleep, frequent night time waking, taking short naps, and needing an adult to settle the child [89]. Research has shown that having an ‘unsettled’ baby or one who did not sleep ‘well’ [89-91] increases the risk of postnatal depression, however, being able to maintain good quality sleep can ameliorate these effects [89-91]. It is considered that poor infant sleeping and self-settling habits are a learned behaviour, and amenable to behavioural change. Therefore, promoting effective infant settling techniques, encouraging good sleeping habits from birth and preventing maternal sleep deprivation may play an important role in helping to reduce postnatal depression.

Numerous research studies have explored the relationship between maternal psychology and postnatal depression. These studies have found that being nervy, angry, shy or introverted, and lacking in assertiveness or confidence, are aspects of maternal psychology that are correlated with postnatal depression [87,89-91]. However, as infant and maternal temperaments are interactive, it is very difficult to establish causal pathways of postnatal depression. As already discussed, having an infant who is perceived as problematic or a poor sleeper can adversely impact on the relationship between mother and child, and increase the risk of postnatal depression. Moreover, relationship interactions between the mother and her partner must also be considered, as difficult marital relationships, partners who are unsupportive, controlling or critical, and domestic violence are also linked to postnatal depression [87,90]. Lack of social support has also been found to be associated with postnatal depression [91].

Scope exists for more research in the area of postnatal depression to more fully determine the complex pathways and risk factors for postnatal depression, and the development of responsive and effective early intervention and support programs. This review found very little research focused particularly on depression during antenatal stages of motherhood. It is recommended that this be an area of future research.

### Depression among women aged 32–64 years

Middle-age has been defined as being between 45 and 64 years of age [92]. Whilst the literature included in the current review provided some information specific to middle-aged women and depression, age categories varied across studies, with the average age of middle-aged women being 37 years. It is noteworthy that of the three age groups reviewed, middle-age received the least attention from the literature.

In this age group, the prevalence of depression varied from 9.2% [58] to 24% [52], most probably attributable to variations in age groupings and other sample differences between studies. Despite methodological differences, the current review found that middle-aged women appear less prone to depression than younger women, but more prone to depressive symptoms than older women [5]. Women aged 45 to 49 years were the most likely of all age groups to be admitted to hospital for a depressive disorder [93]. Moreover, research has found that more females aged 60 years and over attempted and completed suicide compared to younger women [94]. Suicide is 4.4 times higher in females when compared to the general population at older ages and 6.6 times higher among older women who have been in prior contact with mental health services [94]. As depression is a very robust predictor of suicide in older age groups [94], more research into the severity of depressive symptoms at different ages is warranted.

Depression in middle-age shares similar correlations to those occurring among younger women, for example sole motherhood [54,55], lower socio-economic status [95], a history of childhood abuse [57], or history of intimate partner abuse and domestic violence [54,55]. Although some studies suggest that by mid-age many women may no longer be experiencing domestic violence, the psychological impacts of domestic violence or abuse can be long lasting [54,55]. In one study, 39% of women who had experienced any type of abuse felt that the abuse continued to have a negative impact on their lives [54,55].

A significant life stage event that occurs in middle-age is menopause. The current review found that those women who have experienced surgical menopause are at increased risk of depression, compared to those women who experience natural menopause [52]. Additionally, those women who experience a longer menopause transition have higher depressed mood than those women who are postmenopausal [52]. Moreover, the use of hormone therapies made no difference in level of depressive symptoms [52].

Other contributing factors and life stage events correlated with depression in middle-age are having prior pre-menstruation problems, negative attitudes toward ageing and menopause [52], life transitions out of relationships (separation, divorce), children leaving home, and the existence or onset of health problems.

The impact of physical activity on depression was also examined in the current review. Brown et al.’s [60] study of physical activity and depressive symptoms in middle-aged women concluded that physical activity is an important mediator of depression, with a representative sample showing a clear dose–response relationship between physical activity and psychological health [60]. Noticeably in the current review, there were fewer investigations concerned with weight and exercise for women in the middle-age group than there were for younger women. However, this may be an important avenue of enquiry that could provide significant insights into reducing and preventing depression in middle-age.

In the current review, the lack of literature focusing on depression among middle-age women highlights the need for further research and investigation, and the development of responsive intervention and treatment programs for women in the 45 and over age group, particularly given the greater incidence of hospitalisations for depression, and relatively high risk of suicide compared to women of other ages.

### Depression among women aged 64–93 years

The average age of women included in this section was 70 years. The current review found that older women are least likely to experience depression; however, depression among older women can impact substantially due to limited social and family networks and less robust coping mechanisms [27]. Moreover, older women with depression may be less inclined to seek help due to generational stoicism, lack of understanding about depression, and fears about stigma or shame attributed to mental illness [27]. It is also important to note that feelings of shame or stigma may preclude older women from disclosing depressive symptoms in a research context, and therefore any research findings may be skewed. Prevalence estimates of depression among older women in this review vary considerably due to differing sampling compositions, from as low as 1.77% [6], to 34.7% [96] of the representative samples. It is important to note that prevalence rates may vary depending on the setting (i.e. aged care facility, independent living and so on) and also the age of the older person. It is worth noting as part of this discussion that more recent literature suggests that while there is generally a lower prevalence of depression in older age there is an increase in psychological distress in the older stages of life, after 80 years of age [97]. This discussion point requires further investigation and highlights the importance of measuring and understanding depression for women in this older age group.

Among women aged over 60 years, those most at risk of being hospitalised for depression were aged 70–79 years. Moreover, women in this age group are more likely to be hospitalised for depression than women aged in their early twenties [98]. These findings highlight the importance of further research examining severity of depression at different ages of the life course, as mentioned in the previous results section.

Among older women, it is important to note that those residing in Australia but born overseas experience higher prevalence rates of depression than their Australian-born counterparts [61], and this is discussed further in the specific topic section. In common with other age groups, for women aged 64–93 years, smoking and lower levels of physical activity were associated with depression [67,68,95]. Physical and chronic ill health [99] and anxiety [64] were found to be comorbid with depression among older women, and these findings reflect research among younger women. However, older women present additional correlates of depression that may be considered characteristic of this age group, namely, falls and injuries [25], pain [100], functional decline and loss of preferred activities [96], increased dependence on others [99], poor dental health and concerns over appearance of teeth, gums or mouth, and denture problems [53]. Quine and Morrell’s [53] study found that 34.4% of older women reporting problems with oral health or dentures had felt depressed in the previous four weeks. Given that poor oral health or ill-fitting dentures are able to be remedied through increased funding and access to oral health services, this is one area of social health policy that could have a significant impact on improving quality of life and reducing depression among older Australian women.

In the current review, being in receipt of a government pension was also associated with greater prevalence of depression among older women [58,95], suggesting a link between lower economic status and depression. Older women’s reliance on government benefits may be attributed to widowhood, lack of opportunity to accrue superannuation or savings across the life course, disrupted work histories due to family, marital or caring responsibilities, and lower wages generally [58]. Targeted social policies may help to alleviate financial insecurity among older women in Australia, and in turn reduce the burden of depressive illness in this age group. Further research is warranted therefore, to inform more responsive policy formation and development in this area.

The current review identified substantial gaps in research for this age group. In addition to those areas already discussed, and compared to research undertaken among young and middle-aged women, there is insufficient research on the experiences of abuse and depression among older Australian women. It has been suggested elsewhere in this paper that older women may choose not to disclose depressive illness or symptomatology due to stigma or shame, and therefore prevalence rates and research in this area may be incomplete. Despite the appearance of some lower prevalence rates of depression among older women, high rates of hospital admissions for depression among women aged 70–79 years, and the high rates of depression found in some clinical [101] and aged care settings [99] warrants further investigation. Further, the current review indicates more research is needed to fully understand the progress of depressive symptomatology over time and the life course.

### Depression in specific populations

#### Depression among Indigenous Australian women

In 2009, Indigenous Australians accounted for 2.5% of the overall Australian population, of which an estimated 6% identified as Torres Strait Islander populations [97]. In the current review, few studies of depression among Indigenous peoples were identified. Only four studies were found to meet the inclusion criteria for this review [24,50,84,102]; and these studies utilised small sample sizes, ranging in size from 51 [50] to 106 [102] Aboriginal and Torres Strait Islander women.

Whilst the prevalence of depression among Indigenous peoples is undetermined, it is known that Indigenous Australians have 1.9 times higher hospitalisation rates for care involving mental and behavioural disorders than non-Indigenous Australians [1,103]. Anxiety and depression are the foremost health problems reported by Indigenous women in Australia [103]. Several small studies have reported very high rates of depression for Indigenous women, for example, Deemal [24]. Also, studies indicate that Indigenous women appear to experience depression at higher rates than non-Indigenous women, for example, Butler et al.’s [50] study found that 29% of female Indigenous prisoners were experiencing depression (compared to 18% of non-Indigenous female prisoners). A number of correlates were found to be related to depression among Indigenous women, such as unemployment, smoking or having a partner who smoked cigarettes, physical abuse, low coping skills, anxiety, caring for other people’s children [24], and cannabis use [102]; however, the small sample sizes precluded statistical analyses, and the results are not generalisable.

It is important to note that identifying the presence of depression among Indigenous peoples is complicated by the use of translated instruments. For example, Campbell et al. [84] used the Townsville Aboriginal Islander Health Service (TAIHS) and Mount Isa (MTI) translated versions of the Edinburgh Postnatal Depression Scale (EPDS), which in comparison to the standard version, identified higher rates of postnatal depression among a sample of 210 Indigenous women who had given birth [84]. While indicating greater sensitivity, this difference was not significant. Moreover, the researchers noted that the study was limited by the low number of women participants.

In terms of future research, the current review has highlighted the lack of knowledge about the prevalence and correlates of depression among Indigenous Australian women. Importantly, any future research must address the need for culturally appropriate measures and identification of depression and postnatal depression among Indigenous people, whilst displaying a high commitment towards cultural sensitivity and awareness.

#### Depression among culturally and linguistically diverse women

Thirteen studies that met the inclusion criteria for examining depression among women from culturally and linguistically diverse backgrounds were identified. The research included was limited, and many of the findings have been previously referred to, however the main findings will be reiterated here.

Overall, the studies in the current review indicated that having a background other than Australian and living in Australia was associated with depression among Asian Australian high/secondary school students [74], Filipina women [83], Vietnamese and Turkish new mothers [85], oncology outpatients [63], and older people seeking help at a memory clinic [61]. Additionally, absence of social support, lower English proficiency, and being under 25 years of age was significantly associated with depression among new mothers [83,85]. In the current review, the limited number of articles meeting the inclusion criteria and the small sample study sizes in general preclude the drawing of conclusions regarding prevalence or correlations of depression. There is a clear need therefore for further research in this area. Moreover, in common with Indigenous populations, it is important to emphasise that any research with people from culturally and linguistically diverse backgrounds must be conducted in a culturally appropriate and sensitive manner.

#### Rurality and depression among Australian women

Much contention exists over the role of rurality in mental health outcomes, with different studies presenting conflicting prevalence rates and correlations. For example, it is often assumed urban living presents a risk factor for the incidence of depression, whilst rural areas are thought to provide more socially stable, cohesive, and supportive environments. Conversely, other studies have identified higher prevalence rates of depression in rural environments [49,92]. One study suggests rurality presents a risk factor due to typical predictors of depression such as isolation and poverty being exacerbated by rural environments [100]. Whilst urban and rural environments can play an influential role in the prevalence rates of depression for women, the literature in the current review suggests that other factors such as poverty, unemployment, being female, lower socio-economic class, substance misuse, a history of childhood sexual abuse, poor social networks or low perceived social support, an adverse life event in the prior 12 months, size of primary support group, and marital status have a more profound influence on the prevalence of depression for women living in both rural and urban communities [24,59,88].

One difficulty identified in the review literature surrounding geography and assessing prevalence and correlates of depression among Australian women is that of measurement. The ability to measure and define what is rural, remote, urban or metropolitan areas is a complex task that may or may not take into account proximity to the nearest metropolitan area, access to health services, and population density. Some research studies in the current review clearly delineated and defined their geographic terms (for example, GISCA. (2012). About ARIA+ (Accessibility/Remoteness Index of Australia). Retrieved 20/09, 2012, from http://www.adelaide.edu.au/apmrc/research/projects/category/aria.html. However, in other studies geographical locations were not defined, and this is likely to contribute to discrepancies between the reported results and conclusions. More intensive research is needed to alleviate the lack of research in this area, and to more closely determine to what extent rurality influences the prevalence and correlates of depression among Australian women. Moreover, particular attention should be placed on developing common understandings and definitions of geographical measurement to resolve the differences in geographical measurement between studies, and increase the accuracy and generalisability of research findings.

## Discussion

The current systematic literature review has identified 111 articles published between January 1999 to January 2010 that specifically focus on the prevalence and correlates of depression among Australian females aged over 12 years. The current review found that the prevalence of depression in Australia is more common among females, and this trend holds evident despite the differences in depression measurement tools or definitions used in some studies. Moreover, the current review identified a broad range of correlates that vary according to age, socio-economic status, relationship status, Indigenous status, cultural and linguistic diversity, parity, adverse life events including childhood sexual abuse and physical abuse, physical illness and chronic disease, physical inactivity, previous mental health problems, and a range of age and life-stage specific correlates.

### Limitations

The current review is subject to a number of limitations. Publication bias may occur when well-resourced researchers, with track records, are more likely to have their work accepted for publication or identified for the review, compared to less experienced researchers. Moreover, the current review excluded papers that related to depression, but not specifically prevalence or correlates. Therefore, information relating to depression per se may have been excluded. Accordingly, the recommendations that follow are made within the scope of the included literature and in view of these limitations.

### Clinical and policy implications

Throughout this review several recurring factors were identified that appeared to mitigate the impact of risk factors for depression. Similar (or the same) factors appeared to be preventative of depression. Social support, physical activity and higher socio-economic status were associated with decreased risk of depression [31,32,39,40].

These three factors might be useful in both the design of policy and clinical interventions. For example, policies that support those most at risk in pursuing education or employment might result in increased socio-economic status and therefore a decrease in risk of depression. Further, the facilitation of physical activity through government subsidised programs, for example Measure Up [7], could provide both an avenue for better physical health and an opportunity to improve social networks, as well as preventing depression or reducing the incidence of depression among those most at risk.

For practitioners, awareness that women who have experienced abuse, divorce, sole motherhood or chronic physical health (cardiovascular disease, obesity or smoking, poor physical activity) are most at risk of depression is vitally important. In the context of complex physical and social problems, primary care providers should be supported in providing mental health assessments to those most at risk. The Better Access scheme goes some way to providing the support in Australia [3] but time constraints interfere with the ability of practitioners to implement such schemes. On a more positive note, findings for social support and physical activity point the way to how effective interventions might be designed. This report has reviewed a vast number of articles available on this topic and has been provide insights into clinical interventions and necessary academic research.

### Implications for future research

Despite the breadth of the 111 articles selected for inclusion in this review, noticeable gaps in the literature were observed. In the current review articles were excluded if analyses did not distinguish between males and females. In such cases, the lack of gendered analyses precludes the identification of important risk factors and correlates particular to males and females. It is important therefore, that future research considers depression as a gendered experience, and as such, warrants analyses that distinguish between males and females.

It is further noted that wherever possible, depression should be distinguished from other affective and psychological disorders. In the current review, some studies reported on affective disorders, but not specifically depression, whilst other studies treated anxiety and depression as a single construct. This lack of consensus between studies makes it difficult to draw accurate conclusions, and therefore more consistent measurements and definitions of depression are needed.

The current review revealed a noticeable lack of clarity about the relationship between reduced likelihood of depression and cohort or age effects. As women in all age groups experience depression at very different rates and at varying degrees of severity, more information is needed to inform targeted intervention and prevention strategies across the life course. Moreover, associations between age, life stage, life events, demographics, health behaviour, chronic illness, and life transitions (for example, into and out of relationships, or widowhood) and depression, suggest there are complex pathways that precede depression and a variety of consequences that occur subsequent to depression, some of which may exacerbate existing conditions. A comprehensive examination of the pathways into and the consequences of depression are needed to illuminate causal factors in depression, and most importantly, reveal mechanisms that may optimally assist with recovery from depression. Furthermore, the cumulative impact of multiple stressors has yet to be assessed. Further longitudinal research should be undertaken to clarify the nature of these age related differences, and an audit of available longitudinal data undertaken to assist development of plans for analysing these data where possible. In the current review, several longitudinal studies were identified [16-23].

The current review also further proposes that the feasibility of linking population survey data with administrative datasets to examine the progression of depression over time be investigated. Whilst hospital separation results indicated the highest rate of hospitalisation for depression occurred among middle-aged women [93], other population surveys have identified the highest rates of depression as occurring among young women [104]. These data suggest there is more to be learned about the progression and severity of depression over time and across the life course. Linking survey based data with administrative datasets such as hospital separations data offers potential avenues for further investigation.

A dearth of research relating to depression and rurality was also noticeable in the current review, in part because area of residence was not the focus of population based studies, even though datasets often include geographical area as a variable. It is recommended therefore, that existing data be examined to determine the differences, if any, in depression between rural and urban areas. As mentioned above, the possible linking of survey based data to administrative datasets may help to illuminate vital information in this regard.

Although there was a reasonable coverage in the literature of depression among young and older women, there was a noticeable lack of literature in the current review that examined depression in middle-age. In addition to menopause, a number of significant events typically occur in middle-age, including life transitions (separation, divorce, and subsequent marriage), children leaving home, and the onset of chronic health conditions. With middle-age women identified at most risk of hospitalisation for depression, it is vitally important that depression among middle-age women be prioritised for further research to address existing gaps in research literature, and to inform public health policies and initiatives.

Further to those already mentioned, the current review identified a lack of research literature in a number of areas. Namely, research concerning depression among Indigenous and culturally and linguistically diverse women was scarce, or where identified, involved small sample sizes. Moreover, in the current review, no articles were found that examined depression among lesbian, transgender or intersex women. Appropriate research plans should be undertaken to resolve these gaps in current knowledge, and bring to light important information about depression among Indigenous, culturally and linguistically diverse, and lesbian, transgender, and intersex women in Australia. Moreover, any research undertakings must necessarily involve varying levels of consultation with the women concerned, in order to establish culturally appropriate and sensitive research methods and protocols.

In addition to these recommendations, a number of areas were identified in the current review as being in need of further research, many of which relate to the imperative to increase understanding of the factors that underlie certain findings. For example, studies have shown associations between body image, weight problems and depression; however greater insights into the factors that lead to body image and weight problems could inform and optimise intervention strategies, and accordingly, more targeted and in-depth studies on body image, weight problems, and the effects of skin problems and depression among women aged 12 to 60 years are indicated. Moreover, the current review recommends that further, more focused investigations be undertaken in the following areas of research: age at first pregnancy and depression; pregnancy loss and depression; demographic profiling of women most susceptible to postnatal depression; the impact of abuse on older women; and depression among older women living in residential aged care facilities.

## Conclusion

The final recommendation of the current review takes account of the limitations inherent in a review of this type. The results and recommendations of the current review are naturally limited by the scope of the investigation’s aims and the inclusion or exclusion criteria applied to articles during the literature search. Any research that is undertaken is also limited by the research that precedes it and the ideas, theories, interests, and knowledge of the researchers involved; it is necessary therefore to unlock those avenues of exploration that may not be readily discernible. Indeed, women who have experienced or are experiencing depression are ideally able to provide valuable insights and knowledge about their experiences of depression and thus reliably inform further research. The validity of women’s personal experience therefore, should not be discounted. Rather, the authors of the current review recommend that an exploratory study be undertaken with women who have experienced or are experiencing depression, to identify and inform areas of future research and practice.

## Competing interests

The authors declares that they have no competing interest.

## Authors’ contributions

DL, JEB, JLR and JMB designed the study and wrote the protocol. *JLR and JMB managed the literature searches and reviews. The original report commissioned by beyondblue was produced by DL, JMB, JR, and JEB of the Priority Research Centre for Gender, Health and Ageing (PRCGHA) at the University of Newcastle. This systematic review article has been written and edited by JLR, CC, DL, JEB. All authors contributed to and have approved the final manuscript.

## Supplementary Material

Additional file 1: Table S1 List of Measurement Instruments used in the Reviewed Studies. **Table S2.** Preliminary search: Keywords (simple) search terms. **Table S3.** Sources and number of citations obtained. **Table S4.** Health behaviour and depression in Australian women. **Table S5.** Depression among young women aged up to 32 years. **Table S6.** Depression in the pregnancy-related period. **Table S7.** Depression among women aged 32-64 years. **Table S8.** Depression among women aged 64-93 years. **Table S9.** Depression among Indigenous Australian women. **Table S10.** Depression among Culturally and Linguistically Diverse women. **Table S11.** Rurality and depression among Australian women. Click here for file
