# Supplementary material for: Prevalence and correlates of depression among Australian women: a systematic literature review, January 1999- January 2010
Source: BMC Res Notes. 2013 Oct 21;6:424. doi: 10.1186/1756-0500-6-424 (PMC3827921; doi:10.1186/1756-0500-6-424)
Supplement: Additional file 1: Table S1 — List of Measurement Instruments used in the Reviewed Studies. Table S2. Preliminary search: Keywords (simple) search terms. Table S3. Sources and number of citations obtained. Table S4. Health behaviour and depression in Australian women. Table S5. Depression among young women aged up to 32 years. Table S6. Depression in the pregnancy-related period. Table S7. Depression among women aged 32-64 years. Table S8. Depression among women aged 64-93 years. Table S9. Depression among Indigenous Australian women. Table S10. Depression among Culturally and Linguistically Diverse women. Table S11. Rurality and depression among Australian women. [file 1756-0500-6-424-S1.docx]

**Prevalence and Correlates of Depression among Australian Women: A Systematic Literature Review, January 1999- January 2010.**

Jane L. Rich ^a, c *^, Jennifer M. Byrne ^a^, Cassie Curryer ^a^, Julie E. Byles ^a,b,c^, & Deborah Loxton ^a,c^

**please note that all table numbers have now changed.

**Additional file 1**

**List of Tables:**

S1 List of measurement instruments used in the Reviewed Studies.

S2 Preliminary search: Keywords (simple) search terms.

S3 Sources and number of citations obtained.

S4 Health behaviour and depression in Australian women.

S5 Depression among young women aged up to 32 years.

S6 Depression in the pregnancy-related period.

S7 Depression among women aged 32-64 years.

S8 Depression among women aged 64-93 years.

S9 Depression among Indigenous Australian women.

S10 Depression among Culturally and Linguistically Diverse women.

S11 Rurality and depression among Australian women.

# Table S1: List of Measurement Instruments used in the Reviewed Studies.

| **Measurement Instrument Abbreviation** | **Measurement Instrument Full Name** | **Reference details (author, publisher and/or website).** |
| --- | --- | --- |
| **AQoL** | **Assessment of Quality of Life** | Hawthorne, G., J. Richardson, et al. (1999). The Assessment of Quality of Life (AQoL) instrument: a psychometric measure of health-related quality of life. Qual Life Res, 8(3): 209-24.  [http://www.buseco.monash.edu.au/centres/che/pubs/tr12.pdf](http://idacc.healthbase.info/%20http:/www.buseco.monash.edu.au/centres/che/pubs/tr12.pdf) |
| **AUDIT** | **Alcohol Use Disorders Identification Test** | Saunders, J.B., Aasland, O.G., Babor, T.F., de la Fuente, J.R. and Grant, M. (1993). Development of the Alcohol Use Disorders Identification Test (AUDIT): WHO collaborative project on early detection of persons with harmful alcohol consumption. Addiction, 88, 791-804.  <http://whqlibdoc.who.int/hq/2001/who_msd_msb_01.6a.pdf> |
| **BAI** | **Beck Anxiety Inventory** | Beck, Aaron T. and Robert A. Steer. (1993). Beck Anxiety Inventory Manual. San Antonio, TX: The Psychological Corporation Harcourt Brace & Company, 1993.  <http://www.pearsonpsychcorp.com.au/productdetails/39/1/11>  <http://www.acf.hhs.gov/programs/opre/ehs/perf_measures/reports/resourcesmeasuring/res_meas_phib.html> |
| **BDI** | **Beck Depression Inventory, version 2** | Beck, Aaron T., Gregory K. Brown, and Robert A. Steer. (2000). Beck Depression Inventory-II (BDI-II). San Antonio, TX: The Psychological Corporation, 1996. Beck, Aaron T. Beck InterpreTrak. San Antonio, TX: The Psychological Corporation, 2000.  [http://www.swin.edu.au/victims/resources/assessment/affect/bdi.html](http://idacc.healthbase.info/%20http:/www.swin.edu.au/victims/resources/assessment/affect/bdi.html) [http://www.ibogaine.org/graphics/3639b1c_23.pdf](http://idacc.healthbase.info/%20http:/www.ibogaine.org/graphics/3639b1c_23.pdf) |
| **CAMCOG** | **The Cambridge Cognitive Examination for Mental Disorders of the Elderly** | Roth, M., E. Tym, et al. (1986)  CAMDEX: a standardized instrument for the diagnosis of mental disorders in the elderly with special reference to the early detection of dementia. British Journal of Psychiatry, 149: 698-709.  <http://bjp.rcpsych.org/cgi/content/abstract/149/6/698> |
| **CES-D** | **Centre for Epidemiological Studies Depression Scale** | Radloff, LS. (1977). The CES-D Scale: A Self-Report Depression Scale for Research in the General Population. Applied Psychological Measurement, 1(3): 385-401.  <http://www.acf.hhs.gov/programs/opre/ehs/perf_measures/reports/resources_measuring/res_meas_phid.html> |
| **CES-D-10** | **Centre for Epidemiologic Studies Short Depression Scale** | F. J. Kohout, L. F. Berkman, D. A. Evans, and J. Cornoni-Huntley. (1993). Two shorter forms of the CES-D (Center for Epidemiological Studies Depression) depression symptoms index. Journal of Aging Health, 5(2):179-93. |
| **CIDI** | **Composite International Diagnostic Interview** | World Health Organization. (1993). The ICD-10 Classification of Mental and Behavioural Disorders: Diagnostic Criteria for Research. WHO, Geneva.  Robins, Lee N., John Wing, Hans Ulrich Wittchen, John E. Helzer, Thomas F. Babor, Jay Burke, Anne Farmer, Assen Jablenski, Roy Pickens, Darrel A. Regier, Norman Sartorius, Leland H. Towle. (1988). The Composite International Diagnostic Interview: An Epidemiologic Instrument Suitable for Use in Conjunction With Different Diagnostic Systems and in Different Cultures. Arch Gen Psychiatry, 45: 1069-1077.  World Health Organization. (1977). Composite International Diagnostic Interview (CIDI), Core Version 2.1, Interviewer’s Manual. World Health Organization, January 1997. <http://www.acf.hhs.gov/programs/opre/ehs/perf_measures/reports/resources_measuring/res_meas_phif.html> |
| **CIDI-A** | **Composite International Diagnostic Interview - automated presentation** | <http://www.crufad.org/index.php/clinician-services/cidi> |
| **CIS-R** | **Clinical Interview Schedule** | Lewis G, Pelosi AJ, Araya RC, Dunn G. (1992). Measuring psychiatric disorder in the community: a standardized assessment for use by lay interviewers. Psychol Med, 22:465–486. |
| **CSDD** | **Cornell Scale for Depression in Dementia** | Alexopoulos GA, Abrams RC, Young RC & Shamoian CA. (1988). Cornell scale for depression in dementia. Biol Psych, 23:271-284.  <http://www.health.gov.au/internet/main/publishing.nsf/Content/ageing-rescare-natframe.htm~ageing-rescare-natframe08.htm> |
| **DASS** | **Depression Anxiety Stress** | Lovibond, S.H. & Lovibond, P.F. (1995).  *Manual for the Depression Anxiety Stress Scales*. (2nd. Ed.) Sydney: Psychology Foundation.  <http://www2.psy.unsw.edu.au/groups/dass//> |
| **DSM-IV** | **Diagnostic and Statistical Manual of Mental Disorders Fourth Edition** | American Psychiatric Association. (1994). Diagnostic and Statistical Manual of Mental Disorders (DSM-IV), 4th edn. American Psychiatric Press, Washington. |
| **DSSI** | **The Delusion Symptoms States Inventory** | [Bedford](http://www.sciencedirect.com/science?_ob=RedirectURL&_method=outwardLink&_partnerName=27983&_origin=article&_zone=art_page&_linkType=scopusAuthorDocuments&_targetURL=http%3A%2F%2Fwww.scopus.com%2Fscopus%2Finward%2Fauthor.url%3FpartnerID%3D10%26rel%3D3.0.0%26sortField%3Dcited%26sortOrder%3Dasc%26author%3DBedford,%2520A.%26authorID%3D7005936384%26md5%3D97742db0837ea982b2e6fe49a53d331c&_acct=C000047922&_version=1&_userid=915767&md5=42ab877c871bf663cef2931c4f5776c2), [Deary](http://www.sciencedirect.com/science?_ob=RedirectURL&_method=outwardLink&_partnerName=27983&_origin=article&_zone=art_page&_linkType=scopusAuthorDocuments&_targetURL=http%3A%2F%2Fwww.scopus.com%2Fscopus%2Finward%2Fauthor.url%3FpartnerID%3D10%26rel%3D3.0.0%26sortField%3Dcited%26sortOrder%3Dasc%26author%3DDeary,%2520I.%2520J.%26authorID%3D36045816000%26md5%3Dd59784854b8fec8f093b5231faf0b615&_acct=C000047922&_version=1&_userid=915767&md5=ce4d2f29a274282f4966afbd2dcc3982). (1999). The Delusions-Symptoms-States Inventory (DSSI): construction, applications and structural analyses. Personality and Individual Differences, 26(3):397-424. |
| **EPDS** | **Edinburgh Postnatal Depression Scale** | Cox, J.L., Holden, J.M., and Sagovsky, R. (1987). Detection of postnatal depression: Development of the 10-item Edinburgh Postnatal Depression Scale. British Journal of Psychiatry, 150:782-786. |
| **GADS** | **Goldberg Anxiety and Depression Scales** | Goldberg D, Bridges K, Duncan-Jones P, Grayson D. (1988). Detecting anxiety and depression in general medical settings. BMJ, 297: 897-899. |
| **GDS-15** | **The Geriatric Depression Scale** | Yesavage, J. A., Brink, T. L., Rose, T. L., Lum, O., Huang, V., Adey, M. and Leirer, V. O. (1983). Development and validation of a geriatric depression screening scale: A preliminary report. J. Psychiat. Res, 17:37-49.  van Marwijk, H. W. J., Wallace, P., De Bock, G. H., Hernans, J. O., Kptein, A. A. and Mulder, J. D. (1995). Evaluation of the feasibility, reliability and diagnostic value of shortened versions of the geriatric depression scale. Brit. J. Gen. Pract, 45:195-199. (15-item) |
| **GHQ-28** | **General Health Questionnaire** | Goldberg DP. (1978). Manual of the General Health Questionnaire. Windsor, UK: National Foundation for Educational Research (NFER).  Goldberg D, Williams P. (1988). A user's guide to the General Health Questionnaire. Windsor, UK: NFER-Nelson. |
| **HADS** | **Hospital Anxiety and Depression Scale** | Snaith RP. (2003). The Hospital Anxiety and Depression Scale. Health and Quality of Life Outcomes,1:29  <http://www.hqlo.com/content/1/1/29> |
| **HSCL-37** | **Hopkins Symptom Checklist** | Derogatis LR, Lipman RS, Rickels K, Cori L. (1974). The Hopkins symptom checklist (HSCL)—a self-report symptom inventory. Behav Sci, 19: 1–15. |
| **ICD – 10** | **International Statistical Classification of Diseases and Health Related Problems, version 10** | World Health Organization. (1993). The ICD-10 Classification of Mental and Behavioural Disorders: Diagnostic Criteria for Research. WHO, Geneva. |
| **K-10** | **Kessler Psychological Distress Scale** | Kessler and Mroczek. (1994). School of Survey Research Center of the Institute for Social Research. University of Michigan. |
| **MHI-5** | **The 5-item Mental Health Subscale** | Subscale of the SF-36.  http://www.sf-36.org/ |
| **MMPI** | **The Minnesota Multiphasic Personality Inventory** | <http://psychcentral.com/lib/2011/minnesota-multiphasic-personality-inventory-mmpi/> |
| **PD-Q4+** | **Personality Diagnostic Questionnaire** | <http://www.pdq4.com/> |
| **PHQ-9** | **Patient Health Questionnaire** | <http://www.depression-primarycare.org/clinicians/toolkits/materials/forms/phq9/> |
| **PRIME-MD** | **Primary Care Evaluation of Mental Disorders** | Spitzer RL, Williams JB, Kroenke K, et al. Utility of a new procedure for diagnosing mental disorders in primary care: the PRIME-MD 1000 study. JAMA. 1994;272(22):1749–1756 |
| **PVPS** | **Phan Vietnamese Psychiatric Scale** | Phan, Steel, Silove. (2004). An Ethnographically Derived Measure of Anxiety, Depression and Somatization: The Phan Vietnamese Psychiatric Scale. Transcultural Psychiatry, Vol 41 no.2. |
| **RADS** | **Reynolds Adolescent Depression scale** | <http://www.sigmaassessmentsystems.com/assessments/rads.asp> |
| **SAD** | **Seasonal Affective Disorder Scale** | <http://priory.com/psych/SAD.htm> |
| **SCID-I/P** | **Structured Clinical Interview for DSM-IV –** | <http://www.scid4.org/faq/research_version.html> |
| **SF-12** | **Short Form Health Survey** | <http://www.sf-36.org/tools/sf12.shtml> |
| **SF-36** | **Medical Outcome Short Form Health Survey** | http://www.sf-36.org/ |
| **SMFQ** | **Short Mood and Feelings Questionnaire** | Angold A, Costello EJ, Messer SC, Pickles A, Winder F, Silver D. (1995). Development of a short questionnaire for use in epidemiological studies of depression in children and adolescents. International Journal of Methods in Psychiatric Research, 5:237–249. |

**Where direct web links were not easily procurable, citations were noted in their stead*

# Table S2. Preliminary search: Keywords (simple) search terms.

| Subject area | Search terms |
| --- | --- |
| Depression | ‘depressi*’ or ‘dysthym*’ or ‘major depression’ or postnatal depression’ or ‘postpartum depression’ or ‘affective disorder*’ or ‘mood disorder*’. |
| Australia | Search terms were linked with AND ‘Australia*’ as the aim of the project was to focus on Australian data. |
| Prevalence and Correlates | ‘prevalence’ or ‘incidence’ or ‘relat*’ or ‘correlat*’ or associate*’ or ‘risk factor*’ or comorbidit” |
| Women | ‘Wom*n*’ or ‘female*’ or ‘girl*’ AND ‘adolescen*’ or ‘aged’ or ‘older’ or ‘adult’ or ‘middle aged’ or ‘age span’. |
| Indigenous peoples and cultural and linguistically diverse groups | ‘oceanic ancestry groups’ or ‘ethnic group*’ (Medline only)^[[1]](#footnote-1)^, or ‘Indigenous’ or ‘minority group*’ or (Aboriginal and Torres Strait Islander), or ‘multicultural’ or ‘migrant*’ or ‘non-English speaking’ or ‘(racial and ethnic group*)’, (PsychInfo only) or ‘cross cultural’ or ‘immigrant*’ or ‘aborigin*’ or ‘(culturally or linguistically diverse)’^[[2]](#footnote-2)^. Multicultural and Indigenous terms were combined with depression, Australia and gender terms in separate searches, and were then added to the main search. |
| Additional searches | An additional search was performed with the terms ‘therap*’, or ‘treatment’ or ‘complementary’ or ‘evidence’ or ‘evidence based’ or ‘self-help’ or ‘(St Johns Wort)’ or ‘antidepress*’ or ‘psychotherap*’ or ‘guideline*’ or ‘recommendation*’ or ‘(complementary or alternative medicine*)’ which was joined with AND to the main search terms previously described to ensure relevancy. Seven key researcher names were also used as search terms. |

***Table S3. Sources and number of citations obtained.***

| **Source** | **No. of Citations** |
| --- | --- |
| Medline | 823 |
| PsychInfo | 111 |
| SCOPUS | 529 |
| Cinhal | 43 |
| Cochrane | 29 |
| Informit | 233 |
| Author searches | 120 |
| Subtotal | 1888 |
| No. Duplicates (removed) | 617 |
| **Total** | 1271 |

# Table S4. Health behaviour and depression in Australian women.

| **Reference** | **Sample Characteristics** | **Prevalence** | **Main Findings** |
| --- | --- | --- | --- |
| Alati, R., Lawlor, D. A., et al. (2005). "Is there really a 'J-shaped' curve in the association between alcohol consumption and symptoms of depression and anxiety? Findings from the Mater University Study of Pregnancy and its outcomes." Addiction 100(5): 643-651. | Prospective cohort study of women (N = 4,527) who received antenatal care at Mater Misericordiae Hospital in Brisbane between 1981 and 1984 who have provided follow-up data over the subsequent 14-year period. | Depression measured by the Delusions-Symptoms-States Inventory (DSSI) indicated that 9.4% of the total sample had depression. | Significant relationships were found between alcohol intake and depression and anxiety, and may vary across different stages of the life course for women. |
| Australian Institute of Health and Welfare (2006). Chronic diseases and associated risk factors in Australia, 2006. Australia: 96p. | Updated statistics on chronic diseases and their associated risk factors in Australia and differences in chronic diseases and their risk factors across geographical areas, socioeconomic status and Indigenous status. |  | Reports relationship between depression and cardiovascular disease (CVD) and also physical activity, obesity and smoking. |
| Baines, S., Powers, J., et al. (2007). "How does the health and well-being of young Australian vegetarian and semi-vegetarian women compare with non-vegetarians?" Public Health Nutrition 10(5): 436-442. | Cross-sectional data analysis of the Australian Longitudinal Study on Women’s Health (ALSWH) data in 2000, 9,113 women aged 22-27 years participated in the study. |  | Semi-vegetarian and vegetarian women had poorer mental health, with 21-22% reporting depression compared with 15% of non-vegetarians. |
| Ball, K., Burton, N. W., et al. (2009). "A prospective study of overweight, physical activity, and depressive symptoms in young women." Obesity 17(1): 66-71 | 6,677 young adult women aged 22–27 years in 2000, participating in the ALSWH. | More than 25% of the sample had depressive symptoms in 2003, compared with 29% in 2000. Depression measured using the CESD-10. | The presence of depressive symptoms in 2000 was predictive of depressive symptoms in 2003. Mean Center for Epidemiologic Studies Depression Scale (CESD-10) scores in the healthy weight and overweight Body Mass Index (BMI) categories decreased with increasing physical activity. For the obese and underweight BMI categories, there was no consistent downward trend in CESD-10 scores with higher levels of physical activity. Mean depression scores were higher for women in the obese BMI category than the healthy weight category, regardless of physical activity level. |
| Bottomley, K. L., Lancaster, S. J., et al. (2008). "The association between depressive symptoms and smoking in pregnant adolescents." Psychology Health & Medicine 13(5): 574-582. | 81 pregnant adolescents aged 13 – 20 years participated in the study. |  | 30% of the pregnant adolescents were at risk for depression at the first data collection (trimester 1 or 2) and 31% were at risk of depression at the second data collection (trimester 3). Smokers were more likely to be at risk of depression than non-smokers. Depression was measured using the EPDS. |
| Brown, W. J., Ford, J. H., et al. (2005). "Prospective study of physical activity and depressive symptoms in middle-aged women." American Journal of Preventive Medicine 29(4): 265-272. | 9,207 women born between 1946 and 1951 (aged 45-50 years in 1996), who were participants of the Australian Longitudinal Survey on Women’s Health (ALSWH). Reports on an analysis of (ALSWH) data collected in 1996, 1998, and 2001. |  | A clear and significant inverse association between physical activity and depressive symptoms was found. |
| Cassidy, K., Kotynia-English, R., et al. (2004). "Association between lifestyle factors and mental health measures among community-dwelling older women." Australian & New Zealand Journal of Psychiatry 38(11-12): 940-947. | Cross-sectional survey of 270 women aged 70 or over living in the community. |  | Physically active women were half as likely to be depressed (Beck Depression Inventory (BDI) score ≥ 10) when compared to their inactive counterparts. Having ever smoked more than 20 cigarettes per day was associated with increased risk of depression. |
| Darby, A., Hay, P., et al. (2007). "Disordered eating behaviours and cognitions in young women with obesity: relationship with psychological status." International Journal of Obesity 31(5): 876-882. | 4, 891 women aged 18-24 years from the community. |  | Body mass Index (BMI) did not correlate with depression. |
| France, C., Lee, C., et al. (2004). "Correlates of depressive symptoms in a representative sample of young Australian women." Australian Psychologist 39(3): 228-237. | A nationally representative sample of 9,333 Australian women aged 22-27 years who participate in the ALSWH. | Approximately 30% of these young women indicated that they were experiencing depressive symptoms. Depression was measured using the CESD-10. | After adjusting for age and rurality of residence, depressive symptoms were significantly associated with low income, low educational level, unemployment; not being in a relationship.; frequent visits to doctors and medical specialists; a higher number of physical symptoms and diagnosed conditions; illicit drug use, smoking tobacco and using alcohol; and lower exercise status. |
| Jacka, F. N., Pasco, J. A., et al. (2004). "Dietary omega-3 fatty acids and depression in a community sample." Nutritional Neuroscience 7(2): 101-106. | Age-stratified, population-based sample of women (N = 755, aged 23-97 years). | The 12-month prevalence of depression among this sample was 12.85%. A self-report questionnaire based on DSM-IV criteria was utilised to measure depression. | Women who were depressed were younger, more likely to smoke, and weighed more than non-depressed women. No difference in omega-3 essential polyunsaturated fatty acids (PUFA) intake was detected between the depressed and non-depressed groups. |
| Jacka, F. N., Pasco, J. A., et al. (2007). "Self-reported depression and cardiovascular risk factors in a community sample of women." Psychosomatics 48(1): 54-59. | 755 women aged 23-97 years were randomly sampled from the community. | 145 (19.2%) women reported having a lifetime history of depression. A self-report questionnaire was used to measure depression. | Women with a history of smoking or angina were more likely to report a lifetime history of depression than women who had not smoked or experienced angina. |
| Kenardy, J., Brown, W. J., et al. (2001). "Dieting and health in young Australian women." European Eating Disorders Review 9(4): 242-254. | 14,686 Australian women aged 18-23 years who participate in the Australian Longitudinal Study on Women’s Health (ALSWH). |  | High frequency of dieting and earlier onset of dieting was associated with poorer mental health (including depression). |
| Lee, C. (1999). "Health habits and psychological well-being among young, middle-aged and older Australian women." British Journal of Health Psychology 4(Part 4): 301-314. | Questionnaire responses from a nationally representative sample of 612 women in three age groups (18-23, 45-50, 70-75 years). |  | Women who did not exercise were more likely than women who do exercise to experience higher levels of depression. Smoking and unhealthy weight were also associated with depression. The youngest cohort (18-23 years) was most likely to experience psychological distress. |
| Lee, K., Clough, A., et al. (2008). "Heavy cannabis use and depressive symptoms in three Aboriginal communities in Arnhem Land, Northern Territory." Medical Journal of Australia 188(10): 605-608. | 106 Aboriginal men (N = 57, 54%) and women (N = 49, 46%) in remote Arnhem Land, NT, aged 13-42 years (Mean age females 25.6 years). Approximately half of the sample (N = 50) were randomly selected from patient lists in health clinics, and additional numbers were opportunistically recruited by Aboriginal health workers. | 31% of females and 18% males scored in the moderate -severe range for depression. Depression was measured using a modified version of the PHQ-9. | Heavy cannabis users were 4 times more likely to report moderate-severe depression compared with light and non-cannabis users (OR 4.1, 95% CI 1.3-13.4) |
| Rey, J. M., Sawyer, M. G., et al. (2001). "Depression among Australian adolescents." Medical Journal of Australia 175(1): 19-23. | 1,490 adolescents (female N = 687) aged 13-17 years and their main caregiver who participated in the National Survey of Mental Health and Wellbeing Study. | For all adolescents (including males) the prevalence of depression was 5.2%. Adolescents completed self-report questionnaires and parents were interviewed using a lay-administered, structured psychiatric interview and several questionnaires. | Adolescent-reported depression was associated with increased suicide plans and attempts in the previous year, use of marijuana 10 or more times in the previous month, having conduct disorder, and use of school support services. Three per cent of depressed adolescents had been treated with antidepressants. |
| Williams, L. J., Pasco, J. A., et al. (2009). "Lifetime psychiatric disorders and body composition: a population-based study." Journal of Affective Disorders 118(1-3): 173-179. | 979 women aged 20-93 years, randomly selected from south-eastern Australia. | 28.6% of the sample was identified as having a lifetime history of depression. Depression was measured using the SCID-I/NP. In measurement instruments table, SCID-I/P. | A lifetime history of depression was associated with being overweight or obese, being younger, taller, current or past cigarette smoker, higher energy intake. Those with a lifetime history of depression were also more likely to use psychotropic medications. |

# Table S5. Depression among young women aged up to 32 years.

| **Reference** | **Sample Characteristics** | **Prevalence** | **Main Findings** |
| --- | --- | --- | --- |
| Al Mamun, A., Cramb, S., et al. (2007). "Adolescents' perceived weight associated with depression in young adulthood: A longitudinal study." Obesity 15(12): 3097-3105. | 1,802 participants (at age 21 years) from the Mater University of Queensland Study of Pregnancy and Its Outcomes. |  | Perceptions of being overweight during adolescence are a significant risk factor for depression in young women. |
| Baines, S., Powers, J., et al. (2007). "How does the health and well-being of young Australian vegetarian and semi-vegetarian women compare with non-vegetarians?" Public Health Nutrition 10(5): 436-442. | Cross-sectional data analysis of the Australian Longitudinal Study on Women’s Health (ALSWH) data in 2000, 9,113 women aged 22-27 years participated in the study. |  | Semi-vegetarian and vegetarian women had poorer mental health, with 21-22% reporting depression compared with 15% of non-vegetarians. |
| Ball, K., Burton, N. W., et al. (2009). "A prospective study of overweight, physical activity, and depressive symptoms in young women." Obesity 17(1): 66-71 | 6,677 young adult women aged 22–27 years in 2000, participating in the ALSWH. | More than 25% of the sample had depressive symptoms in 2003, compared with 29% in 2000. Depression was measured using the CESD-10. | The presence of depressive symptoms in 2000 was predictive of depressive symptoms in 2003. Mean Center for Epidemiologic Studies Depression Scale (CESD-10) scores in the healthy weight and overweight Body Mass Index (BMI) categories decreased with increasing physical activity. For the obese and underweight BMI categories, there was no consistent downward trend in CESD-10 scores with higher levels of physical activity. Mean depression scores were higher for women in the obese BMI category than the healthy weight category, regardless of physical activity level. |
| Bond, L., Toumbourou, J. W., et al. (2005). "Individual, Family, School, and Community Risk and Protective Factors for Depressive Symptoms in Adolescents: A Comparison of Risk Profiles for Substance Use and Depressive Symptoms." Prevention Science 6(2): 73-88. | 8,984 students (females N = 4,653) were recruited from three High School (HS) Years in Victoria. Participants Mean ages :12 years old (HS Year 7), 14 years old (HS Year 9) and 16 years old (HS Year 11). | The prevalence rate of depression was significantly higher among female (22%) compared with male students. This study examines the relationship between adolescent depressive symptoms and risk and protective factors identified for substance use. A questionnaire, developed to measure these factors in a young person's community, family, school, peer group, and individual characteristics for substance use, was used to assess associations with self-reported depressive symptoms. |  |
| Bottomley, K. L., Lancaster, S. J., et al. (2008). "The association between depressive symptoms and smoking in pregnant adolescents." Psychology Health & Medicine 13(5): 574-582. | 81 pregnant adolescents aged 13 – 20 years participated in the study. |  | 30% of the pregnant adolescents were at risk for depression at the first data collection (trimester 1 or 2) and 31% were at risk of depression at the second data collection (trimester 3). Smokers were more likely to be at risk of depression than non-smokers. Depression was measured using the EPDS. |
| Boyd, C. P., Kostanski, M., et al. (2000). "Prevalence of anxiety and depression in Australian adolescents: comparisons with worldwide data." Journal of Genetic Psychology 161(4): 479-492. | The combined sample comprised 1,299 (675 female) adolescents aged 11 – 18 years old, randomly selected from metropolitan and country schools in Melbourne. | More females than males reported depression (18.8% vs. 9.3%), and females had significantly higher levels of anxiety than males (17.5% vs. 8.5%). Depression was measured using the RADS. | Overall, 14.2% of adolescents were identified as depressed and 13.2% were identified as having anxiety. The prevalence of depression and anxiety differed across countries and cultures. No significant differences for age were found. |
| Butterworth, P., Rodgers, B., et al. (2009). "Financial hardship, socio-economic position and depression: results from the PATH Through Life Survey." Social Science & Medicine 69(2): 229-237. | Two waves of data from The Path Through Life Study were used. Three cohorts of women (N = 6,715) aged 24-28 years, 44-48 years and 64-68 years were involved. | In the younger cohort, prevalence in wave 1 for depression was 12.2%, for the mid cohort 9.2%, and the older cohort 3.3%. At wave two prevalence rates for depression for the younger cohort was 12.2%, mid cohort 9.0%, and older cohort 2.6%. Depression was measured using the Goldberg Depression Scale |  |
| Campbell, A., Hayes, B., et al. (2008). "Aboriginal and Torres Strait Islander women's experience when interacting with the Edinburgh Postnatal Depression Scale: a brief note." Australian Journal of Rural Health 16(3): 124-131. | 210 Indigenous women (at antenatal or postnatal stage) from Townsville (N =181, 86.2%), Mt Isa (N = 14, 6.7%), and Yapatjarra (N = 11, 5.2%) participated. The women’s ages ranged from ≤ 20 years (N=33, 15.7%), 21 to 25 years (N = 96, 45.7%), 26 to 30 years (N = 35, 16.7%), 31 to 35 years (N = 33, 15.7%), 36-40 years (N = 11, 5.2%) and ≤40 years (N = 2, 1%). | Used translated versions of the Edinburgh Postnatal Depression Scale (EPDS) which when compared to the standard version, identified no significant difference in rates of depression at postnatal review (28% using the translated versions compared to 24.6% of non-translated EPDS). | The ‘translations’ of the EPDS demonstrated a high level of reliability. The ‘translations’ and the standard EPDS both identified high rates of Indigenous women at risk of depression. |
| Darby, A., Hay, P., et al. (2007). "Disordered eating behaviours and cognitions in young women with obesity: relationship with psychological status." International Journal of Obesity 31(5): 876-882. | 4, 891 women aged 18-24 years from the community. |  | Body mass Index (BMI) did not correlate with depression. |
| Deemal, A. (2001). ""What choice do we have, there's no place for us to go": Young Women's Emotional and Mental Health Study." Aboriginal and Islander Health Worker Journal 25(5): 28-31. | A sample of 52 Indigenous women completed surveys and participated in focus groups. (Mean age = 22.94 years). | The prevalence of reported depression was found to be 53.8%. | Depression was associated with unemployment, smoking, physical abuse, low coping skills, no place to relax or unwind, anxiety and distress, caring for other people’s children and having partners who smoked cigarettes. |
| Dingle, K., Alati, R., et al. (2008). "Pregnancy loss and psychiatric disorders in young women: an Australian birth cohort study." British Journal of Psychiatry 193(6): 455-460. | A sample of 1,223 women of a cohort born between 1981 and 1984 were assessed at age 21 years for psychiatric and substance abuse disorders and lifetime pregnancy history. |  | Abortion and miscarriage were associated with experiencing affective disorders including major depression, dysthymia and bipolar disorder. |
| Donald, M. and Dower, J. (2002). "Risk and protective factors for depressive symptomatology among a community sample of adolescents and young adults." Australian and New Zealand Journal of Public Health 26(6): 555-562. | 3,082 adolescents and young adults (females N = 1,710) aged 15-24 years from Queensland. |  | Depression was significantly associated with parental problems, sexual abuse, sexual identity conflict, financial difficulty, relationship break-up, being bullied, scholastic failure, introversion, a higher level of neuroticism and aggressive behaviour. |
| Donald, M. and Dower, J. (2002). "Risk and protective factors for depressive symptomatology among a community sample of adolescents and young adults." Australian and New Zealand Journal of Public Health 26(6): 555-562. | Case-control design. A clinical sample of 18–24 year olds recruited via the emergency department hospital following a suicide attempt (N=95) were compared to a sample of 18–24 year olds who participated in a population-based survey (N=380). |  | Risk factors for medically serious suicide attempts included early school leaving, parental divorce (males only), and distress due to problems with parents (females only). |
| Donald, M., Dower, J., et al. (2001). "Prevalence of adverse life events, depression and suicidal thoughts and behaviour among a community sample of young people aged 15-24 years." Australian and New Zealand Journal of Public Health 25(5): 426-432. | Cross-sectional household survey of 3,092 participants aged 15-24 years of age. | Females reported significantly higher rates of depression than males (OR= 1.72, 95% CI 1.47 - 2.01). A cross-sectional household survey, using telephone recruitment followed by a postal pencil-and-paper questionnaire. | Females were significantly more likely than males to report having experienced suicidal thoughts and to have attempted suicide. |
| Duke, J. M., Sibbritt, D. W., et al. (2007). "Is there an association between the use of oral contraception and depressive symptoms in young Australian women?" Contraception 75(1): 27-31. | Data from the Australian Longitudinal Survey on Women’s Health (ALSWH) was analysed. Responses from Survey 2 (N = 8,636) when women were aged between 22-27 years, and Survey 3 (N =7,489) when women were aged 25-30 years, were included in the analysis. | 2,488 (28.8%) of the women responding to Survey 2 and 1,943 (25.9%) of Survey 3 respondents reported depressive symptoms. | Oral contraception was used by 61.9% of women in Survey 2 with 56.1% in Survey 3. In Survey 3 almost one quarter (23.3%) of oral contraceptive users reported depressive symptoms, while 30.3% of non-users reported depressive symptoms. The odds of a non-user experiencing depressive symptoms was 1.43 (95%CI = 1.28-1.58) times that of an oral contraceptive user. |
| France, C., Lee, C., et al. (2004). "Correlates of depressive symptoms in a representative sample of young Australian women." Australian Psychologist 39(3): 228-237. | A nationally representative sample of 9,333 Australian women aged 22-27 years who participate in the ALSWH. | Approximately 30% of these young women indicated that they were experiencing depressive symptoms. Depression was measured using the CESD-10. | After adjusting for age and rurality of residence, depressive symptoms were significantly associated with low income, low educational level, unemployment; not being in a relationship.; frequent visits to doctors and medical specialists; a higher number of physical symptoms and diagnosed conditions; illicit drug use, smoking tobacco and using alcohol; and lower exercise status. |
| Heaven, P. C. and Goldstein, M. (2001). "Parental influences and mental health among some Australian youth: crosscultural analysis." Australian Journal of Psychology 53(3): 170-175. | 202 high school students: 92 Australian Anglos (53 Females, 39 males), 110 of Asian origin (69 females and 41 males) including students of Cambodian, Chinese, or Vietnamese decent and lesser numbers from Philipino, Laotian, Japanese & Taiwanese origin. Participants were aged between 13-18 years (median = 16 years), recruited from three secondary government schools in Western Sydney, Australia. |  | Asian Australians had significantly higher depression scores compared to Anglo Australians, and females had significantly higher levels of depression compared to males. Post hoc analyses showed Anglo females had higher depression and lower self-esteem than Anglo males. |
| Jorm, A. F., Rodgers, B., et al. (1999). "Smoking and mental health: results from a community survey." Medical Journal of Australia 170(2): 74-77. | 2,725 (female N = 1,426) participants aged 18-79 years sampled from the Australian electoral roll. |  | As depression, anxiety and neuroticism were highly correlated the investigators combined depression and anxiety symptoms into a single score and omitted neuroticism. Women with anxiety/depression were 1.4 times more likely to smoke relative to women without these problems. |
| Kenardy, J., Brown, W. J., et al. (2001). "Dieting and health in young Australian women." European Eating Disorders Review 9(4): 242-254. | 14,686 Australian women aged 18-23 years who participate in the Australian Longitudinal Study on Women’s Health (ALSWH). |  | High frequency of dieting and earlier onset of dieting was associated with poorer mental health (including depression). |
| Leach, L. S., Christensen, H., et al. (2008). "Gender differences in depression and anxiety across the adult lifespan: the role of psychosocial mediators." Social Psychiatry & Psychiatric Epidemiology 43(12): 983-998. | Representative community based sample of 7,485 participants from Canberra and Queanbeyan, Australia, in three age groups: 20-24, 40-44 and 60-64 years. | Prevalence of depression in the previous month for women aged 20-24 was 3.18%, 40-44 age group 2.56% and 60-64 age group 1.77%. Depression was measured using the GADS. | Gender differences in depression between men and women were mediated by physical symptoms, physical activity, and psychological and interpersonal factors. |
| Lee, C. (1999). "Health habits and psychological well-being among young, middle-aged and older Australian women." British Journal of Health Psychology 4(Part 4): 301-314. | Questionnaire responses from a nationally representative sample of 612 women in three age groups (18-23, 45-50, 70-75 years). |  | Women who did not exercise were more likely than women who do exercise to experience higher levels of depression. Smoking and unhealthy weight were also associated with depression. The youngest cohort (18-23 years) was most likely to experience psychological distress. |
| Lee, C. and Gramotnev, H. (2007). "Life transitions and mental health in a national cohort of young Australian women." Developmental Psychology 43(4): 877-888. | Transitions among a nationally representative cohort of 7,619 young adult women who participated in the ALSWH Survey 2 (aged 22-27 years in 2000) and Survey 3 (aged 25-30 years in 2003). |  | Transitioning into intimate relationships was associated with improvements to mental health, while reductions in mental health were associated with transitioning to marital separation or divorce. Symptoms increased for women moving out of study or paid work and among those moving into motherhood relative to women who did not experience such transitions. |
| Loxton, D., Mooney, R., et al. (2006). "The psychological health of sole mothers in Australia." Medical Journal of Australia 184(6): 265-268. | Data were analysed from 9,689 women aged 22-27 years and 1, 338 women aged 47-52 years who participate in the ALSWH. |  | Among the younger women, sole mothers were more likely to have experienced suicidal thoughts (odds ratio (OR 2.18, 95% CI 1.45–3.27) and self-harm (OR 3.25, 95% CI 1.97–5.38). Sole mothers were most likely to have used medication for depression (OR 2.75, 95% CI 1.76). Sole mothers were more than twice as likely to have experienced depression, and had significantly poorer psychological health. |
| Lubman, D. I., Allen, N. B., et al. (2007). "The impact of co-occurring mood and anxiety disorders among substance-abusing youth." Journal of Affective Disorders 103(1-3): 105-112. | 100 participants aged 16-22 years old (Mean age = 19.4 yrs; female N = 47), were recruited from two drug treatment centres in Melbourne, Australia. | Among females, lifetime prevalence of Major Depressive Disorder was 53.2% and 31.9% for current Major Depressive Disorder. A structured interview and questionnaires assessing drug use, psychopathology, risk-taking behaviours and quality of life were administered. | Participants with major depressive disorder (MDD) were more likely than those without MMD to have a higher number of comorbid disorders. Participants with MMD also reported more substance-related problems and a poorer quality of life. |
| Magin, P., Sibbritt, D., et al. (2009). "The relationship between psychiatric illnesses and skin disease: a longitudinal analysis of young Australian women." Archives of Dermatology 145(8): 896-902. | ALSWH data from 6,630 women aged 21-26 years in 2000, who completed three surveys conducted in 2000, 2003, and 2006. | Depression prevalence in 2000 was 8.7%, 2003 8.1%, and 2006 7.9%. Depression was measured using the CES-D. | Depression symptoms and stress was significantly associated with skin problems. |
| Martin, G., Bergen, H. A., et al. (2004). "Sexual abuse and suicidality: gender differences in a large community sample of adolescents." Child Abuse & Neglect 28(5): 491-503. | Community sample of 2,485 adolescents (Mean age 14 years, female N = 1,106) recruited from schools in South Australia. | 33.7% females scored greater than 16 on the Center for Epidemiologic Studies Depression Scale (CES-D), indicating a high potential for clinical depression. | In adolescent females, sexual abuse was associated with suicidality. Depressive symptomatology, hopelessness, and family functioning appeared to mediate the relationship. |
| McKelvey, R., Pfaff, J., et al. (2001). "The relationship between chief complaints, psychological distress, and suicidal ideation in 15-24-year-old patients presenting to general practitioners." Medical Journal of Australia 175(10): 550-552. | 3,242 consecutive patients aged 15-24 years old presenting to 247 participating general practitioners during a specified six-week period. Participants were predominantly females (66%). |  | Females were more likely than males to report symptoms of depression that exceeded cut points for the presence of depression. |
| Migliorini, C. E., New, P. W., et al. (2009). "Comparison of depression, anxiety and stress in persons with traumatic and non-traumatic post-acute spinal cord injury." Spinal Cord 47(11): 783-788. | 443 community dwelling adults (28% female, Mean age 52 years) with a spinal cord injury were recruited from a spinal cord injury registry. |  | Among all participants (including males) there was a 3% decrease in the likelihood of depression with every year post injury. Lower socio-economic status was also associated with depression. Depression was measured using the DASS-21. |
| Mills, K. L., Teesson, M., et al. (2004). "Young people with heroin dependence: findings from the Australian Treatment Outcome Study (ATOS)." Journal of Substance Abuse Treatment 27(1): 67-73. | A cohort of 210 young Australians (44% female) aged between 18 and 24 years, who were participants in the Australian Treatment Outcome Study. |  | Compared with males, females were twice as likely to have current depression and/or a history of suicide attempts. |
| Osborne, R. H., Elsworth, G. R., et al. (2003). "Age-specific norms and determinants of anxiety and depression in 731 women with breast cancer recruited through a population-based cancer registry." European Journal of Cancer 39(6): 755-762. | Population-based hospital sample of 731 women with breast cancer (aged 23-60 years). | The prevalence of probable psychological morbidity due to depression was 3%. Depression was measured using the HADS. | There was no clear pattern of risk factors for depression in this sample. |
| Patton, G. C., Coffey, C., et al. (2001). "Parental 'affectionless control' in adolescent depressive disorder." Social Psychiatry & Psychiatric Epidemiology 36(10): 475-480. | A two-phase study of early onset depression conducted during the course of a six-wave, 3 year study of adolescent health in 2,032 Australian secondary school students. Participants meeting the criteria for depressive episode between waves 2 to 6 were selected for second phase assessment. |  | Low maternal and paternal care was associated with a two- to three-fold higher rate of depressive disorder. |
| Patton, G. C., Olsson, C., et al. (2008). "Predicting female depression across puberty: a two-nation longitudinal study." Journal of the American Academy of Child & Adolescent Psychiatry 47(12): 1424-1432. | Three wave longitudinal cross-cultural study of male and female Secondary School students aged 10 – 15 years in Washington, USA (N = 2,885 first wave; Mean age females = 12.6 years) and Victoria, Australia (N = 2,884 first wave; Mean age females = 12.4 yrs). | For females, the overall rates of high depressive symptoms were 26% (95% CI 24-28) at baseline. Higher rates of female depressive symptoms were found at the subsequent two waves. Depression was measured using the SMFQ. | For females, being in the later stages of puberty, family conflict and bullying were associated with the presence of depression and depressive symptoms. |
| Phillips, J., Sharpe, L., et al. (2007). "Rates of depressive and anxiety disorders in a residential mother- infant unit for unsettled infants." Australian and New Zealand Journal of Psychiatry 41(10): 836-842. | 160 women (Mean = 31.4 years) with infants aged 2 weeks to 12 months recruited from a residential family care facility in Australia. | 25.1% of the sample met criteria for a current diagnosis of depression, 31.7% had met criteria for depression since becoming pregnant. Depression was measured using the EPDS and the SCID-I/P (Research version). | Anxiety was comorbid with depression.  There are substantially higher rates of major depression in women presenting to residential services for unsettled infant behaviour compared to women from community postnatal samples. |
| Rey, J. M., Sawyer, M. G., et al. (2001). "Depression among Australian adolescents." Medical Journal of Australia 175(1): 19-23. | 1,490 adolescents (female N = 687) aged 13-17 years and their main caregiver who participated in the National Survey of Mental Health and Wellbeing Study. | For all adolescents (including males) the prevalence of depression was 5.2%. Adolescents completed self-report questionnaires and parents were interviewed using a lay-administered, structured psychiatric interview and several questionnaires. | Adolescent-reported depression was associated with increased suicide plans and attempts in the previous year, use of marijuana 10 or more times in the previous month, having conduct disorder, and use of school support services. Three per cent of depressed adolescents had been treated with antidepressants. |
| Wilhelm, K., Mitchell, P., et al. (2003). "Prevalence and correlates of DSM-IV major depression in an Australian national survey." Journal of Affective Disorders 75(2): 155-162. | Data from the National Survey of Mental Health and Well-being in Australia. 10,641 participants aged between 18-75 years. | For females, the total prevalence rate of depression in the previous 12 months was 3.9%. Highest prevalence rate of 5.2% was found in middle-aged females. Using data from the National Survey of Mental Health and Well-being, and compares the results with other national studies | Being unemployed, smoking, having a medical condition, being in midlife, previously married, and being female were correlated with current major depression. |
| Williams, L. J., Pasco, J. A., et al. (2009). "Lifetime psychiatric disorders and body composition: a population-based study." Journal of Affective Disorders 118(1-3): 173-179. | 979 women aged 20-93 years, randomly selected from south-eastern Australia. | 28.6% of the sample was identified as having a lifetime history of depression. Depression was measured using the SCID-I/NP. | A lifetime history of depression was associated with being overweight or obese, being younger, taller, current or past cigarette smoker, higher energy intake. Those with a lifetime history of depression were also more likely to use psychotropic medications. |

# Table S6. Depression in the pregnancy-related period.

| **Reference** | **Sample Characteristics** | **Prevalence** | **Main Findings** |
| --- | --- | --- | --- |
| Bilszta, J. L., Gu, Y. Z., et al. (2008). "A geographic comparison of the prevalence and risk factors for postnatal depression in an Australian population." Australian & New Zealand Journal of Public Health 32(5): 424-430. | Urban (N = 908) and rural (N = 1,058) women (Mean 31 years old) attending perinatal health services in Victoria. | No significant difference found in prevalence of postnatal depression between urban and rural communities. Depression was measured using the EPDS. |  |
| Boyce, P. M., Johnstone, S. J., et al. (2000). "Functioning and well-being at 24 weeks postpartum of women with postnatal depression." Archives of Women's Mental Health 3(3): 91-97. | Prospective cohort design. 424 postpartum women (Mean 28 years old ), recruited from Nepean, Cowra, Dudley and Orange Hospitals (New South Wales, Australia) completed questionnaires assessing functioning and well-being (SF-36), and postnatal depression (Edinburgh Postnatal Depression Scale) (EPDS). |  | Compared with women who did not have postnatal depression, women with postnatal depression were more likely to experience role limitations due to physical and emotional problems, lower levels of social functioning, more bodily pain, and lower mental health and vitality (as measured by the SF-36). |
| Brooks, J., Nathan, E., et al. (2009). "Tailoring screening protocols for perinatal depression: prevalence of high risk across obstetric services in Western Australia." Archives of Women's Mental Health 12(2): 105-112. | 4, 838 women (Mean 29 years old) recruited from 3 hospitals and a family birthing centre were screened during pregnancy and within 12 months postpartum. | Of the 3, 853 women who completed the Edinburgh Postnatal Depression Scale (EPDS) postnatally, 6% were considered at high risk of postnatal depression. |  |
| Brown, S., Bruinsma, F., et al. (2004). "Early discharge: no evidence of adverse outcomes in three consecutive population-based Australian surveys of recent mothers, conducted in 1989, 1994 and 2000." Paediatric and Perinatal Epidemiology 18(3): 202-213. | 2,952 women aged 15 years and over completed mailed questionnaires from maternity hospitals and home birth practitioners in Victoria. (Age range for the entire sample not specified.) |  | Unadjusted OR showed that women who left hospital within 48 hours were significantly more likely to be depressed at 5–6 months postpartum than women who stayed in hospital ≥ 5 days (18.2% compared to 12.9%). However, after adjusting for obstetric and social factors, no association between length of stay and depression scores at 5–7 months postpartum was found. Depression was measured using the EPDS. |
| Buist, A., Austin, M., et al. (2008). "Postnatal mental health of women giving birth in Australia 2002- 2004: findings from the *beyondblue* National Postnatal Depression Program." Australian and New Zealand Journal of Psychiatry 42(1): 66-73. | 12,361 postnatal women recruited from 43 health services (public and private hospitals) across Australia. Mean age 30.3 years, with 78.8% of women were born in Australia. | 7.5% of the sample scored > 12 on the Edinburgh Postnatal Depression Scale (EPDS) (indicative of being at risk for postnatal depression). The highest percentage of women scoring EPDS >12 were in Queensland and South Australia (both 10.2%); Western Australia had the lowest percentage of women in the at risk category (5.6%). | Women recruited from private health services in Western Australia were less likely than women recruited from the public health service to be at high risk of postnatal depression. |
| Durkin, S., Milgrom, J., et al. (2004). "Metropolitan regional differences in primary health care of postnatal depression." The Australian Journal of Advanced Nursing 21(3): 20-27. | 213 women (Mean age 31 years) recruited from antenatal clinics at four major public hospitals in Melbourne covering two socioeconomic regions, one eastern (more affluent) and one western (less affluent) (according to the Australian Bureau of Statistics, ABS). |  | Mothers from a less affluent area reported fewer postnatal depressive symptoms than mothers from a more affluent area. |
| Edwards, B., C. Galletly, et al. (2008). "Antenatal Psychosocial Risk Factors and Depression Among Women Living in Socioeconomically Disadvantaged Suburbs in Adelaide, South Australia." Australian and New Zealand Journal of Psychiatry 42(1): 45-50. | 421 women completed the Antenatal Psychosocial Questionnaire (APQ) and the Edinburgh Postnatal Depression Scale (EPDS). | Logistic regression analysis was used to identify psychosocial risk factors predictive of EPDS caseness. 88% of women endorsed at least one psychosocial risk factor. 35.6% had been  abused as children, 34.9% had suffered recent major life stresses, 24.5% had thoughts of  self harm, 8% admitted to recently hitting or hurting someone in anger and 5.6% had been  victims of violence since becoming pregnant. | The incidence of depression, measured  using an EPDS cut-off score of 10 or more, was 29.7%. |
| Fisher, J., Feekery, C., et al. (2002). "Nature, severity and correlates of psychological distress in women admitted to a private mother-baby unit." Journal of Paediatrics and Child Health 38(2): 140-145. | A cross-sectional self-report survey of consecutive patients admitted to Masada Private Hospital Mother-Baby Unit, Melbourne (N = 109, Mean age 33.3 years). Mean age of infants was 22 weeks. | 48% of participants scored in the clinical range of depressive symptoms. Depression was measured using the EPDS. | Probable depression was associated with having a partner who was perceived as critical and controlling, a lack of assertiveness and oversensitivity to the opinions of others, as well as having an unsettled baby. |
| Harwood, K., McLean, N., et al. (2007). "First-time mothers' expectations of parenthood: What happens when optimistic expectations are not matched by later experiences?" Developmental Psychology 43(1): 1-12. | 71 first time mothers (Mean age 27.7 years). |  | Depression was more likely for women whose expectations of parenthood before birth were not met by their experiences measured at four months post partum. |
| Hiscock, H. and Wake, M. (2001). "Infant sleep problems and postnatal depression: a community-based study." Pediatrics 107(6): 1317-1322. | 738 mothers completed a survey. | 15% of mothers scored above 12 on the EPDS, indicating probable clinical depression. 18% scored between 10 and 12, indicating possible clinical depression. | Infant sleeping problems were a significant predictor of a depression score >10 (OR 2.88, 95%CI 1.93, 4.31). However, good sleep quality protected against depression associated with infant sleeping problems. |
| Johnstone, S. J., Boyce, P. M., et al. (2001). "Obstetric risk factors for postnatal depression in urban and rural community samples." Australian & New Zealand Journal of Psychiatry 35(1): 69-74. | A prospective study of 490 women from Wentworth and Central Coast regions (NSW) using records from the NSW Midwives Data Collection and information obtained from questionnaires completed 1 week and 8 weeks postpartum. |  | An increased odd of postnatal depression was associated with self-rated nervousness, shyness/self-consciousness, feeling ‘obsessional’, angry, or a worried. Major health problems and arguments with partner were also significantly associated with postnatal depression. History of depression, anxiety, previous postnatal depression, or having a family member with a psychiatric illness also increased the risk of current postnatal depression. |
| Matthey, S., Speyer, J., et al. (2008). "Changes in unsettled infant sleep and maternal mood following admission to a parent craft residential unit." Early Human Development 84(9): 623-629. | Mothers (N = 116, Mean age 31 years) with infants aged three weeks to three years old presenting to a five-day program at an Australian residential parent craft service because of unsettled infant sleep. | 55% of mothers reported depressive symptomatology within the clinical range on admission. Depression was measured using the EPDS and the HADS-Anxiety subscale. | By 5 weeks post-discharge 80% of the women reported significant improvements in maternal mood (depression and anxiety). Emotional health improvements were most often attributed to the improvement in the infant's sleep. |
| McMahon, C., Barnett, B., et al. (2001). "Postnatal depression, anxiety and unsettled infant behaviour." Australian & New Zealand Journal of Psychiatry 35(5): 581-588. | 128 mothers admitted to the residential care unit of a parent craft hospital (Mean age 31 years) were compared to 58 mothers in a demographically matched group recruited from a private obstetric practice (Mean age 32 years). | 36% of residential care mothers compared with 6% of comparison mothers scored above 12 on the Edinburgh Postnatal Depression Scale (EPDS). 62% of mothers in the residential care group met diagnostic criteria for a major depressive episode occurring since birth. | Mothers admitted to the residential care program were more likely than the matched non-residential care mothers to report postnatal depression. |
| McMahon, C., Trapolini, T., et al. (2008). "Maternal state of mind regarding attachment predicts persistence of postnatal depression in the preschool years." Journal of Affective Disorders 107(1-3): 199-203. | A prospective study of 92 mothers of first-born infants recruited from a parent craft hospital at four months postpartum, with follow-up at 4 years after the birth (Mean age 34.9 years). |  | Severity of depressive symptoms at four months and maternal state of mind regarding attachment at one year postpartum were significant predictors of depression at four years postpartum. Women with an insecure state of mind regarding attachment at one year postpartum were more likely to report ongoing depression. |
| Phillips, J., Sharpe, L., et al. (2007). "Rates of depressive and anxiety disorders in a residential mother- infant unit for unsettled infants." Australian and New Zealand Journal of Psychiatry 41(10): 836-842. | 160 women (Mean = 31.4 years) with infants aged 2 weeks to 12 months recruited from a residential family care facility in Australia. | 25.1% of the sample met criteria for a current diagnosis of depression, 31.7% had met criteria for depression since becoming pregnant. Depression was measured using the EPDS. | Anxiety was comorbid with depression.  There are substantially higher rates of major depression in women presenting to residential services for unsettled infant behaviour compared to women from community postnatal samples. |
| Small, R., Lumley, J., et al. (2003). "Cross-cultural experiences of maternal depression: associations and contributing factors for Vietnamese, Turkish and Filipino immigrant women in Victoria, Australia." Ethnicity & Health 8(3): 189-206. | 318 women aged 14 - 41 years from culturally and linguistically diverse backgrounds were recruited from postnatal hospital wards in Victoria, Australia (104 Vietnamese, 107 Filipina and 107 Turkish women). | 9.7% of the Vietnamese women, 28.8% of the Turkish women, and 7.6% of the Filipina women met criteria for depression. | Significant associations with depression included parity, being less than 25, and having spent less time in Australia, migrated to get married, lower English proficiency, limited social support, and having physical health problems and body pain. |
| Thorpe, K. (2007). "Child health nurses supporting parents." Australian Nursing Journal 14(8): 32-37, 39. | 400 women in third trimester of pregnancy and 12 weeks postpartum. 55% were first time mothers. |  | First-time parents had a marginally higher average depression score. Depression was associated with infant temperament, social support and parenting self efficacy (confidence) (statistics not reported). |

# Table S7. Depression among women aged 32-64 years.

| **Reference** | **Sample Characteristics** | **Prevalence** | **Main Findings** |
| --- | --- | --- | --- |
| Australian Institute of Health and Welfare (2006). Chronic diseases and associated risk factors in Australia, 2006. Australia: 96p. | Updated statistics on chronic diseases and their associated risk factors in Australia and differences in chronic diseases and their risk factors across geographical areas, socioeconomic status and Indigenous status. |  | Reports relationship between depression and cardiovascular disease (CVD) and also physical activity, obesity and smoking. |
| Batterham, P. J., Christensen, H., et al. (2009). "Modifiable risk factors predicting major depressive disorder at four year follow-up: a decision tree approach." BMC Psychiatry 9: 75. | 2,105 20-24 year olds, 2,323 40-44 year olds, and 2,177 60-64 year old males and females participated in this study. | Of the 3,383 females participating in the study 3,237 (95.7%) had no major depressive disorder, while 146 (4.3%) reported major depressive disorder. |  |
| Brown, W. J., Ford, J. H., et al. (2005). "Prospective study of physical activity and depressive symptoms in middle-aged women." American Journal of Preventive Medicine 29(4): 265-272. | 9,207 women born between 1946 and 1951 (aged 45-50 years in 1996), who were participants of the Australian Longitudinal Survey on Women’s Health (ALSWH). Reports on an analysis of (ALSWH) data collected in 1996, 1998, and 2001. |  | A clear and significant inverse association between physical activity and depressive symptoms was found, depression was measured using the CESD-10. |
| Bruinsma, F. J., Venn, A. J., et al. (2006). "Concern about tall stature during adolescence and depression in later life." Journal of Affective Disorders 91(2-3): 145-152. | 650 women of tall stature (≥177cms) who were identified from paediatric records as having undergone a height assessment during adolescence, and subsequently not been treated or had been treated, with oestrogen during adolescence to reduce their adult height (Mean age 38.7 years). | The prevalence of major depression in both treated and untreated ‘tall girls’ was high (12-month prevalence: untreated 10.7%, treated 11.2%; lifetime prevalence: untreated 29.4% treated 26.6%). Depression was measured using the CIDI. | Factors significantly  associated with lifetime major depression in this study were self-reported difficulties during adolescence being the reason for seeking a medical assessment of height (OR 2.25, 95% CI 1.4–3.6) and a negative experience of the assessment or treatment procedures (OR 2.04, 95% CI 1.4–3.0). |
| Butterworth, P., Gill, S. C., et al. (2006). "Retirement and mental health: analysis of the Australian National Survey of Mental Health and Wellbeing." Social Science & Medicine 62(5): 1179-1191. | Analysis of data from the National Survey of Mental Health and Wellbeing (1997), a cross-sectional survey of 10,641 Australian adults. The prevalence of depression was analysed in a sub-sample of men (N = 1,928) and women (N = 2,261) aged 45-74 years. | For women, those aged 65–69 and 70–74 had significantly lower rates of depression than those aged 45–49. Depression was measured using the CIDI. | For women, physical health and receiving a government pension or allowance as the main source of income was shown to significantly contribute to poorer mental health. |
| Butterworth, P., Rodgers, B., et al. (2009). "Financial hardship, socio-economic position and depression: results from the PATH Through Life Survey." Social Science & Medicine 69(2): 229-237. | Two waves of data from The Path Through Life Study were used. Three cohorts of women (N = 6,715) aged 24-28 years, 44-48 years and 64-68 years were involved. | In the younger cohort, prevalence in wave 1 for depression was 12.2%, for the mid cohort 9.2%, and the older cohort 3.3%. At wave two prevalence rates for depression for the younger cohort was 12.2%, mid cohort 9.0%, and older cohort 2.6%. Depression was measured using the Goldberg Depression Scale |  |
| Dennerstein, L., Guthrie, J. R., et al. (2004). "A population-based study of depressed mood in middle-aged, Australian-born women." Menopause 11(5): 563-568. | Melbourne Women's Midlife Health Project data from 314 women aged 45-55 years. | Seventy-five women (24%) had a score of 10 or greater on the Center for Epidemiologic Studies Depression Scale (CES-D) and were classified as being depressed. | Women who had experienced a surgical menopause had significantly higher CES-D scores (higher depressogenic symptomatology) than those women who experienced natural menopause. |
| Draper, B. and Low, L.-F. (2009). "Patterns of hospitalisation for depressive and anxiety disorders across the lifespan in Australia." Journal of Affective Disorders 113(1-2): 195-200. | Specialists psychiatric hospital separation data compiled by the Australian Institute of Health and Welfare (AIHW) in Australia's National Hospital Morbidity Database 1998 - 2005. |  | Women who were aged 45-49 years were the most likely of all women to be hospitalised with a depressive disorder. |
| Lawrence, D., Almeida, O. P., et al. (2000). "Suicide and attempted suicide among older adults in Western Australia." Psychological Medicine 30(4): 813-821. | Hospital admissions and mental health service contact records for all suicide attempts and deaths in period 1980-1995 were analysed. |  | More females aged over 60 years attempted and completed suicide compared with females aged less than 60 years of age. Attempted and completed suicides were associated with previous contact with mental health services. |
| Leach, L. S., Christensen, H., et al. (2008). "Gender differences in depression and anxiety across the adult lifespan: the role of psychosocial mediators." Social Psychiatry & Psychiatric Epidemiology 43(12): 983-998. | Representative community based sample of 7,485 participants from Canberra and Queanbeyan, Australia, in three age groups: 20-24, 40-44 and 60-64 years. | Prevalence of depression in the previous month for women aged 20-24 was 3.18%, 40-44 age group 2.56% and 60-64 age group 1.77%. Depression was measured using the GADS. | Gender differences in depression between men and women were mediated by physical symptoms, physical activity, and psychological and interpersonal factors. |
| Lee, C. (1999). "Health habits and psychological well-being among young, middle-aged and older Australian women." British Journal of Health Psychology 4(Part 4): 301-314. | Questionnaire responses from a nationally representative sample of 612 women in three age groups (18-23, 45-50, 70-75 years). |  | Women who did not exercise were more likely than women who do exercise to experience higher levels of depression. Smoking and unhealthy weight were also associated with depression. The youngest cohort (18-23 years) was most likely to experience psychological distress. |
| Loxton, D., Mooney, R., et al. (2006). "The psychological health of sole mothers in Australia." Medical Journal of Australia 184(6): 265-268. | Data were analysed from 9,689 women aged 22-27 years and 1, 338 women aged 47-52 years who participate in the ALSWH. |  | Among the younger women, sole mothers were more likely to have experienced suicidal thoughts (odds ratio (OR 2.18, 95% CI 1.45–3.27) and self-harm (OR 3.25, 95% CI 1.97–5.38). Sole mothers were most likely to have used medication for depression (OR 2.75, 95% CI 1.76). Sole mothers were more than twice as likely to have experienced depression, and had significantly poorer psychological health. |
| Loxton, D., Schofield, M., et al. (2006). "Psychological health in midlife among women who have ever lived with a violent partner or spouse." Journal of Interpersonal Violence 21(8): 1092-1107. | A nationally representative sample of 11,310 women participants (1946-1951 cohort) of the ALSWH. Women were selected on the basis of having indicated at Survey 1 (1996) and/or Survey 2 (1998) (aged 47-52 years) that they had experienced or were currently experiencing violence or abuse. |  | Results indicate that a history of domestic violence is associated with decreased psychological wellbeing and increased depression among middle aged Australian women. |
| McCutcheon, V., Heath, A., et al. (2009). "Accumulation of trauma over time and risk for depression in a twin sample." Psychological Medicine 39(3): 431-441. | Structured diagnostic interviews with 5,266 participants (M = 29.9 years, 55.7% female) from the Australian Twin Register. | 30.8% of females met the lifetime criteria for depression. Histories of depression and trauma were obtained during structured diagnostic interviews. | Assaultive traumatic childhood events had the strongest association with immediate and long-term risk for depression. |
| Osborne, R. H., Elsworth, G. R., et al. (2003). "Age-specific norms and determinants of anxiety and depression in 731 women with breast cancer recruited through a population-based cancer registry." European Journal of Cancer 39(6): 755-762. | Population-based hospital sample of 731 women with breast cancer (aged 23-60 years). | The prevalence of probable psychological morbidity due to depression was 3%. Depression was measured using the HADS. | There was no clear pattern of risk factors for depression in this sample. |
| Wilhelm, K., Mitchell, P., et al. (2003). "Prevalence and correlates of DSM-IV major depression in an Australian national survey." Journal of Affective Disorders 75(2): 155-162. | Data from the National Survey of Mental Health and Well-being in Australia. 10,641 participants aged between 18-75 years. | For females, the total prevalence rate of depression in the previous 12 months was 3.9%. Highest prevalence rate of 5.2% was found in middle-aged females. Depression was measured using the computerised version of the CIDI. | Being unemployed, smoking, having a medical condition, being in mid life, previously married, and being female were correlated with current major depression. |

# Table S8. Depression among women aged 64-93 years.

| **Reference** | **Sample Characteristics** | **Prevalence** | **Main Findings** |
| --- | --- | --- | --- |
| Butterworth, P., Gill, S. C., et al. (2006). "Retirement and mental health: analysis of the Australian National Survey of Mental Health and Wellbeing." Social Science & Medicine 62(5): 1179-1191. | Analysis of data from the National Survey of Mental Health and Wellbeing (1997), a cross-sectional survey of 10,641 Australian adults. The prevalence of depression was analysed in a sub-sample of men (N = 1,928) and women (N = 2,261) aged 45-74 years. | For women, those aged 65–69 and 70–74 had significantly lower rates of depression than those aged 45–49. Depression was measured using the CIDI. | For women, physical health and receiving a government pension or allowance as the main source of income was shown to significantly contribute to poorer mental health. |
| Butterworth, P., Rodgers, B., et al. (2009). "Financial hardship, socio-economic position and depression: results from the PATH Through Life Survey." Social Science & Medicine 69(2): 229-237. | Two waves of data from The Path Through Life Study were used. Three cohorts of women (N = 6,715) aged 24-28 years, 44-48 years and 64-68 years were involved. | In the younger cohort, prevalence in wave 1 for depression was 12.2%, for the mid cohort 9.2%, and the older cohort 3.3%. At wave two prevalence rates for depression for the younger cohort was 12.2%, mid cohort 9.0%, and older cohort 2.6%. Depression was measured using the Goldberg Depression Scale |  |
| Buys, L., Roberto, K. A., et al. (2008). "Prevalence and predictors of depressive symptoms among rural older Australians and Americans." The Australian Journal of Rural Health 16(1): 33-39. | Rural participants (N = 216; female N = 107) aged 65+ years completed a postal survey as part of the Australian Active Ageing Survey (Triple A). |  | Pain significantly predicted depressive symptoms among women. |
| Byers, A. L., van Doorn, C., et al. (2003). "Paternal Attachment as a Risk Factor for Depression in Older Women." Journal of Mental Health and Aging 9(3): 157-169. | Data collected from 487 women aged over 70 years who participated in the Australian Longitudinal Study on Ageing. |  | A linear trend emerged (p=0.04, CI 95% 1.01, 1.59). Results suggest that with every 10 year increase in the magnitude of the younger age of paternal death there is a 27% increase in the odds of depression. |
| Cassidy, K., Kotynia-English, R., et al. (2004). "Association between lifestyle factors and mental health measures among community-dwelling older women." Australian & New Zealand Journal of Psychiatry 38(11-12): 940-947. | Cross-sectional survey of 270 women aged 70 or over living in the community. |  | Physically active women were half as likely to be depressed (Beck Depression Inventory (BDI) score ≥ 10) when compared to their inactive counterparts. Having ever smoked more than 20 cigarettes per day was associated with increased risk of depression. |
| Hammond, A. J., Yu, S., et al. (2008). "Factors associated with persistent risk of depression in older people following discharge from an acute cardiac unit." International Psychogeriatrics 20(4): 738-751. | 191 patients examined at baseline and 1 month post-discharge. Mean age 75 years, females comprised 50% of sample. |  | Gender was not significantly associated with risk of depression (CI 95% 0.6, 2.5). |
| Kerse, N., Flicker, L., et al. (2008). "Falls, depression and antidepressants in later life: a large primary care appraisal." PLoS ONE [Electronic Resource] 3(6): e2423. | Cross-sectional survey of 21,900 community dwelling adults (Mean 71.8 years old; 58.4% female.) |  | Over 60% of women older than 80 years with depression and using antidepressants had fallen or sustained an injury. Depression was measured using the PHQ-9. |
| Koloski, N. A., Smith, N., et al. (2008). "Performance of the Goldberg Anxiety and Depression Scale in older women." Age and Ageing 37(4): 464-467. | Data from the older cohort of women of the Australian Longitudinal Survey on Women’s Health (ALSWH) were examined. Participants were aged between 75 - 80 years at the time of the survey. |  | Anxiety and depression were highly correlated. |
| Lawrence, D., Almeida, O. P., et al. (2000). "Suicide and attempted suicide among older adults in Western Australia." Psychological Medicine 30(4): 813-821. | Hospital admissions and mental health service contact records for all suicide attempts and deaths in period 1980-1995 were analysed. |  | More females aged over 60 years attempted and completed suicide compared with females aged less than 60 years of age. Attempted and completed suicides were associated with previous contact with mental health services. |
| Logiudice, D., Hassett, A., et al. (2001). "Equity of access to a memory clinic in Melbourne? Non-English speaking background attenders are more severely demented and have increased rates of psychiatric disorders." International Journal of Geriatric Psychiatry 16(3): 327-334. | Retrospective analysis of 556 consecutive patients aged over 65 years, from a Melbourne memory clinic. 148 patients (28.8%) were from non-English speaking backgrounds (NESB). |  | Patients from non-English speaking backgrounds (NESB) were more likely to present with a psychiatric disorder than patients from English speaking backgrounds (ESB). No significant differences between males and females found. |
| McCutcheon, V., Heath, A., et al. (2009). "Accumulation of trauma over time and risk for depression in a twin sample." Psychological Medicine 39(3): 431-441. | Structured diagnostic interviews with 5,266 participants (M = 29.9 years, 55.7% female) from the Australian Twin Register. | 30.8% of females met the lifetime criteria for depression. Depression was measured using a structured diagnostic interview. | Assaultive traumatic childhood events had the strongest association with immediate and long-term risk for depression. |
| Migliorini, C. E., New, P. W., et al. (2009). "Comparison of depression, anxiety and stress in persons with traumatic and non-traumatic post-acute spinal cord injury." Spinal Cord 47(11): 783-788. | 443 community dwelling adults (28% female, Mean age 52 years) with a spinal cord injury were recruited from a spinal cord injury registry. |  | Among all participants (including males) there was a 3% decrease in the likelihood of depression with every year post injury. Lower socio-economic status was also associated with depression. Depression was measured using the DASS. |
| O'Connor, D. W., Rosewarne, R., et al. (2001). "Depression in primary care. 1: elderly patients' disclosure of depressive symptoms to their doctors." International Psychogeriatrics 13(3): 359-365. | 1,021 patients (59% female) aged 70+ years recruited through 30 general practices in Melbourne, Australia. |  | 26% of females disclosed depressive symptoms to their GP compared to 17% of males. Depression was measured using the ICD-10. |
| Pfaff, J. J., Almeida, O. P., et al. (2005). "Detecting suicidal ideation in older patients: identifying risk factors within the general practice setting." British Journal of General Practice 55(513): 269-273. | 1,061 patients (57% female) aged 60 - 101 years (M = 72.2, SD = 7.3) attending one of 54 randomly selected Western Australian GPs. 15 consecutive patients from each practice were invited to participate. | 23.8% scored 16 or more on the CES-D. There were no age or sex differences. | Only 5.0% of patients reported psychological distress as the reason for attending the GP, resulting in a greater attention to physical complaints during the vast majority of medical consultations. Over a fifth of those patients presenting for somatic reasons were also experiencing clinically significant depressive symptomatology and 5% acknowledged current suicidal thoughts. |
| Pirkis, J., Pfaff, J., et al. (2009). "The community prevalence of depression in older Australians." Journal of Affective Disorders 115(1-2): 54-61. | 20,226 patients (female N = 12880) aged over 60 years were recruited by their general practitioners. | For females, the age-adjusted prevalence of clinically significant depression was 7.9%. Depression was measured using the PHQ-9. | Around 8% of older Australians are experiencing clinically significant depressive symptoms, and nearly 2% may be experiencing a major depressive episode. Forty two per cent (major depressive episode) and 35% (clinically significant depression) were taking antidepressants. Ten per cent of those with no depression were also taking antidepressants. |
| Quine, S. and Morrell, S. (2009). "Hopelessness, depression and oral health concerns reported by community dwelling older Australians." Community Dental Health 26(3): 177-182. | 8,881 randomly selected community-dwelling participants (56.8% female) aged 65+ years. | 34.4% of female respondents reported ‘feeling depressed’ in the previous 4 weeks. | Concerns about the appearance of teeth, mouth, gum, and dentures was positively correlated with depressive symptoms. |
| Rylands, K. and Rickwood, D. J. (2001). "Ego-integrity versus ego-despair: the effect of "accepting the past" on depression in older women." International Journal of Aging & Human Development 53(1): 75-89. | 73 female residents of a retirement village in Canberra. Mean age 80.6 years. | Level of depression was found to be relatively high in the sample with 30% meeting criteria for depression. | Having difficulty accepting the past, lower levels of social support and increasing levels of physical dependency were all related to increased depression. |
| Snowdon, J., Fleming, R., et al. (2008). "Recognising depression in residential facilities: an Australian challenge." International Journal of Geriatric Psychiatry 23(3): 295-300. | 1,758 residents (females N = 1,242) with a Mean age of 82.1 years were recruited from aged care facilities. | 34.7% of the residents scored 8 or more on the Cornell Scale for Depression in Dementia (CSDD), indicating depression. 41.1% of 1,250 residents tested with the 15-item Geriatric Depression Scale (GDS-15) scored 6 or more, indicating depression. | For all participants (including males) depression was associated with grief over lost abilities including the ability to undertake preferred activities. |
| Trollor, J. N., Anderson, T. M., et al. (2007). "Prevalence of mental disorders in the elderly: The Australian National Mental Health and Well-being Survey." The American Journal of Geriatric Psychiatry 15(6): 455-466. | 1,792 men and women aged over 65 years (female N = 1061) who were participants in the National Mental Health and Well-being Survey in Australia. |  | Females were more likely than males to have experienced an affective disorder. After controlling for socio-demographics, cognitive disorders and physical disorders, the association between gender and affective disorders became non-significant. |
| Wilhelm, K., Mitchell, P., et al. (2003). "Prevalence and correlates of DSM-IV major depression in an Australian national survey." Journal of Affective Disorders 75(2): 155-162. | Data from the National Survey of Mental Health and Well-being in Australia. 10,641 participants aged between 18-75 years. | For females, the total prevalence rate of depression in the previous 12 months was 3.9%. Highest prevalence rate of 5.2% was found in middle-aged females. Depression was measured using the computerised version of the CIDI. | Being unemployed, smoking, having a medical condition, being in mid life, previously married, and being female were correlated with current major depression. |
| Williams, L. J., Pasco, J. A., et al. (2009). "Lifetime psychiatric disorders and body composition: a population-based study." Journal of Affective Disorders 118(1-3): 173-179. | 979 women aged 20-93 years, randomly selected from south-eastern Australia. | 28.6% of the sample was identified as having a lifetime history of depression. Depression was measured using the SCID-I/NP. Table of scales doesn’t have N. | A lifetime history of depression was associated with being overweight or obese, being younger, taller, current or past cigarette smoker, higher energy intake. Those with a lifetime history of depression were also more likely to use psychotropic medications. |

# Table S9. Depression among Indigenous Australian women.

| **Reference** | **Sample Characteristics** | **Prevalence** | **Main Findings** |
| --- | --- | --- | --- |
| Australian Bureau of Statistics and Australian Institute of Health and Welfare (2008). The Health and Welfare of Australia’s Aboriginal and Torres Strait Islander Peoples. Canberra: 320p. | This report presents the latest data on the health, welfare and sociodemographic circumstances of Australia's Indigenous peoples. The estimated resident Indigenous population was 517,200, (2.5% of the total Australian population). | For Indigenous females, the leading specific health problems were anxiety and depression, accounting for 10% of the health burden. |  |
| Australian Institute of Health and Welfare (2008). Indicators for chronic diseases and their determinants, 2008. Australia: 119p. | The report uses findings from other Australian Institute of Health and Welfare (AIHW) reports and data sources to look at favourable and unfavourable trends in chronic disease over time. | No recent national estimates about the prevalence of people with diagnosed depressive disorders in the general population are reported. | Contributing factors for depression including: stressful factors in a person’s environment such as poverty, unemployment, child abuse and exposure to adverse life events (for example, relationship break-ups, trauma and family illness); risk behaviours for example, illicit drug use, alcohol misuse and dependence; eating disorders and excess weight; and diagnosis of chronic diseases such as cardiovascular disease, diabetes, cancer, and rheumatoid arthritis. |
| Butler, T., Allnutt, S., Kariminia, Azar, et al. (2007). "Mental health status of Aboriginal and non-Aboriginal Australian prisoners." Australian and New Zealand Journal of Psychiatry 41(5): 429-435. | 914 prisoners participating in the NSW Inmate Survey. (211 non-Indigenous women and 59 Indigenous women, Mean ages 31.2 and 27 years respectively). | 28.8% of Indigenous women and 17.6% of non-Indigenous women were diagnosed with depression using the CIDI-A. | Indigenous women were more likely to be depressed than non-Indigenous women. |
| Campbell, A., Hayes, B., et al. (2008). "Aboriginal and Torres Strait Islander women's experience when interacting with the Edinburgh Postnatal Depression Scale: a brief note." Australian Journal of Rural Health 16(3): 124-131. | 210 Indigenous women (at antenatal or postnatal stage) from Townsville (N =181, 86.2%), Mt Isa (N = 14, 6.7%), and Yapatjarra (N = 11, 5.2%) participated. The women’s ages ranged from ≤ 20 years (N=33, 15.7%), 21 to 25 years (N = 96, 45.7%), 26 to 30 years (N = 35, 16.7%), 31 to 35 years (N = 33, 15.7%), 36-40 years (N = 11, 5.2%) and ≤40 years (N = 2, 1%). | Used translated versions of the Edinburgh Postnatal Depression Scale (EPDS) which when compared to the standard version, identified no significant difference in rates of depression at postnatal review (28% using the translated versions compared to 24.6% of non-translated EPDS). | The ‘translations’ of the EPDS demonstrated a high level of reliability. The ‘translations’ and the standard EPDS both identified high rates of Indigenous women at risk of depression. |
| Deemal, A. (2001). ""What choice do we have, there's no place for us to go": Young Women's Emotional and Mental Health Study." Aboriginal and Islander Health Worker Journal 25(5): 28-31. | A sample of 52 Indigenous women completed surveys and participated in focus groups. (Mean age = 22.94 years). | The prevalence of reported depression was found to be 53.8%. | Depression was associated with unemployment, smoking, physical abuse, low coping skills, no place to relax or unwind, anxiety and distress, caring for other people’s children and having partners who smoked cigarettes. |
| Lee, K., Clough, A., et al. (2008). "Heavy cannabis use and depressive symptoms in three Aboriginal communities in Arnhem Land, Northern Territory." Medical Journal of Australia 188(10): 605-608. | 106 Aboriginal men (N = 57, 54%) and women (N = 49, 46%) in remote Arnhem Land, NT, aged 13-42 years (Mean age females 25.6 years). Approximately half of the sample (N = 50) were randomly selected from patient lists in health clinics, and additional numbers were opportunistically recruited by Aboriginal health workers. | 31% of females and 18% males scored in the moderate -severe range for depression. Depression was measured using a modified version of the PHQ-9. | Heavy cannabis users were 4 times more likely to report moderate-severe depression compared with light and non-cannabis users (OR 4.1, 95% CI 1.3-13.4) |

# Table S10. Depression among Culturally and Linguistically Diverse women.

| **Reference** | **Sample Characteristics** | **Prevalence** | **Main Findings** |
| --- | --- | --- | --- |
| Alati, R., Najman, J., et al. (2004). "The mental health of Filipino-born women 5 and 14 years after they have given birth in Australia : a longitudinal study." Health Sociology Review 13(2): 145-156. | Longitudinal research on Filipino women who migrated to Australia to marry Australian men (N = 46) compared with Australian women (N = 3,429). Women were interviewed 5 times: first visit to clinic; three to five days postpartum; six months postpartum; five years and 14 years after the birth of their child. |  | Filipina women had smaller social networks compared to Australian women at the time of the birth, and had more symptoms of depression up to 5 years after the birth of the child. However, there were no differences in depressive symptoms between Filipina and Australian born women at 14 years postpartum. |
| Gholizadeh, L., Salamonson, Y., et al. (2009). "Awareness and causal attributions of risk factors for heart disease among immigrant women living in Australia." Journal of Women's Health 18(9): 1385-1393. | Women born in Turkey (N = 17), Iran (N = 18), and Other Arabic countries (N = 20), who were living in metropolitan Sydney, and who had not experienced an acute cardiovascular event, were recruited using the snowballing technique. Bilingual helpers facilitated recruitment. | 24% of the sample had mild to moderate depression and 27% reported severe to very severe depression. Depression was measured using a questionnaire, including investigator-developed instruments and the Depression, Anxiety and Stress Scale. | Psychological distress was associated with lower levels of education, a lack of physical exercise but not with length of time living in Australia. |
| Heaven, P. C. and Goldstein, M. (2001). "Parental influences and mental health among some Australian youth: crosscultural analysis." Australian Journal of Psychology 53(3): 170-175. | 202 high school students: 92 Australian Anglos (53 Females, 39 males), 110 of Asian origin (69 females and 41 males) including students of Cambodian, Chinese, or Vietnamese decent and lesser numbers from Philipino, Laotian, Japanese & Taiwanese origin. Participants were aged between 13-18 years (median = 16 years), recruited from three secondary government schools in Western Sydney, Australia. |  | Asian Australians had significantly higher depression scores compared to Anglo Australians, and females had significantly higher levels of depression compared to males. Post hoc analyses showed Anglo females had higher depression and lower self-esteem than Anglo males. |
| Logiudice, D., Hassett, A., et al. (2001). "Equity of access to a memory clinic in Melbourne? Non-English speaking background attenders are more severely demented and have increased rates of psychiatric disorders." International Journal of Geriatric Psychiatry 16(3): 327-334. | Retrospective analysis of 556 consecutive patients aged over 65 years, from a Melbourne memory clinic. 148 patients (28.8%) were from non-English speaking backgrounds (NESB). |  | Patients from non-English speaking backgrounds (NESB) were more likely to present with a psychiatric disorder than patients from English speaking backgrounds (ESB). No significant differences between males and females found. |
| Pascoe, S., Edelman, S., et al. (2000). "Prevalence of psychological distress and use of support services by cancer patients at Sydney hospitals." Australian and New Zealand Journal of Psychiatry 34(5): 785-791. | 504 oncology cancer outpatients aged 20 to 93 years (median = 62 years) (55% female) from four Sydney hospitals. | Prevalence of depression was 7.1%. No significant effect of gender or age was found. Depression was measured using HADS. | Factors which predicted clinically significant depression were restricted activity, advanced disease, and coming from a non-English speaking background. |
| Schweitzer, R., Melville, F., et al. (2006). "Trauma, post-migration living difficulties, and social support as predictors of psychological adjustment in resettled Sudanese refugees." Australian & New Zealand Journal of Psychiatry 40(2): 179-187. | 63 Sudanese participants (female N = 21) aged over 18 years (Mean age = 34.2 years) in Southeast Queensland were recruited via snowball sampling technique. | 16% of participants were identified as having a major depressive disorder. A semi-structured interview including a questionnaire assessing depression was used. | Women experienced more depressive symptomatology than men. Social support plays a significant role in predicting mental health outcomes. Pre-migration trauma, family status, and gender were also associated with mental health outcomes. |
| Small, R., Lumley, J., et al. (2003). "Cross-cultural experiences of maternal depression: associations and contributing factors for Vietnamese, Turkish and Filipino immigrant women in Victoria, Australia." Ethnicity & Health 8(3): 189-206. | 318 women aged 14 - 41 years from culturally and linguistically diverse backgrounds were recruited from postnatal hospital wards in Victoria, Australia (104 Vietnamese, 107 Filipina and 107 Turkish women). | 9.7% of the Vietnamese women, 28.8% of the Turkish women, and 7.6% of the Filipina women met criteria for depression. | Significant associations with depression included parity, being less than 25, and having spent less time in Australia, migrated to get married, lower English proficiency, limited social support, and having physical health problems and body pain. |
| Steel, Z., Silove, D., et al. (2005). "Mental disorders, disability and health service use amongst Vietnamese refugees and the host Australian population." Acta Psychiatrica Scandinavica 111(4): 300-309. | A household survey of 1,611 Vietnamese aged 18 years and over and living in New South Wales, was compared with national survey data from 7,961 randomly selected Australian-born participants. | Major depression prevalence in Vietnamese females was 2.2%; Australian born females was 8.6%. Depression was measured using the CIDI 2.1 and the MOS SF-12. | Vietnamese with a mental illness reported higher disability but exhibited similar levels of mental health consultation. |
| Tang, G. W., Dennis, S., et al. (2009). "Anxiety and depression in Chinese patients attending an Australian GP clinic." Australian Family Physician 38(7): 552-555. | A cross sectional survey was undertaken with 161 Chinese patients aged over 18 years (70% female, Mean age 47.6 years) attending a general practice in south-western Sydney, NSW, Australia during July 2005. | 15.9% of females reported ever having depression. Depression was measured using theK10 and the SPHERE. | Females were more likely than males to report ever having had depression. There was an association between increased risk of depression or anxiety and reduced occupational status but not social isolation. |

# Table S11. Rurality and depression among Australian women.

| **Reference** | **Sample Characteristics** | **Prevalence** | **Main Findings** |
| --- | --- | --- | --- |
| Australian Institute of Health and Welfare (2006). Chronic diseases and associated risk factors in Australia, 2006. Australia: 96p. | Updated statistics on chronic diseases and their associated risk factors in Australia and differences in chronic diseases and their risk factors across geographical areas, socioeconomic status and Indigenous status. |  | Reports relationship between depression and cardiovascular disease (CVD) and also physical activity, obesity and smoking. |
| Buys, L., Roberto, K. A., et al. (2008). "Prevalence and predictors of depressive symptoms among rural older Australians and Americans." The Australian Journal of Rural Health 16(1): 33-39. | Rural participants (N = 216; female N = 107) aged 65+ years completed a postal survey as part of the Australian Active Ageing Survey (Triple A). |  | Pain significantly predicted depressive symptoms among women. |
| Byers, A. L., van Doorn, C., et al. (2003). "Paternal Attachment as a Risk Factor for Depression in Older Women." Journal of Mental Health and Aging 9(3): 157-169. | Data collected from 487 women aged over 70 years who participated in the Australian Longitudinal Study on Ageing. |  | A linear trend emerged (p=0.04, CI 95% 1.01, 1.59). Results suggest that with every 10 year increase in the magnitude of the younger age of paternal death there is a 27% increase in the odds of depression. |
| Deemal, A. (2001). ""What choice do we have, there's no place for us to go": Young Women's Emotional and Mental Health Study." Aboriginal and Islander Health Worker Journal 25(5): 28-31. | A sample of 52 Indigenous women completed surveys and participated in focus groups. (Mean age = 22.94 years). | The prevalence of reported depression was found to be 53.8%. | Depression was associated with unemployment, smoking, physical abuse, low coping skills, no place to relax or unwind, anxiety and distress, caring for other people’s children and having partners who smoked cigarettes. |
| Johnstone, S. J., Boyce, P. M., et al. (2001). "Obstetric risk factors for postnatal depression in urban and rural community samples." Australian & New Zealand Journal of Psychiatry 35(1): 69-74. | A prospective study of 490 women from Wentworth and Central Coast regions (NSW) using records from the NSW Midwives Data Collection and information obtained from questionnaires completed 1 week and 8 weeks postpartum. |  | An increased odd of postnatal depression was associated with self-rated nervousness, shyness/self-consciousness, feeling ‘obsessional’, angry, or worried. Major health problems and arguments with partner were also significantly associated with postnatal depression. History of depression, anxiety, previous postnatal depression, or having a family member with a psychiatric illness also increased the risk of current postnatal depression. |
| Kilkkinen, A., Kao-Philpot, A., et al. (2007). "Prevalence of psychological distress, anxiety and depression in rural communities in Australia." The Australian Journal of Rural Health 15(2): 114-119. | A cross-sectional survey of 1,536 Australian men and women aged 25-74 years, randomly selected from the Australian electoral roll. |  | There were no gender differences in the prevalence of either mild or moderate to severe depression. |
| Lee, K., Clough, A., et al. (2008). "Heavy cannabis use and depressive symptoms in three Aboriginal communities in Arnhem Land, Northern Territory." Medical Journal of Australia 188(10): 605-608. | 106 Aboriginal men (N = 57, 54%) and women (N = 49, 46%) in remote Arnhem Land, NT, aged 13-42 years (Mean age females 25.6 years). Approximately half of the sample (N = 50) were randomly selected from patient lists in health clinics, and additional numbers were opportunistically recruited by Aboriginal health workers. | 31% of females and 18% males scored in the moderate -severe range for depression. Depression was measured using a modified version of the PHQ-9. | Heavy cannabis users were 4 times more likely to report moderate-severe depression compared with light and non-cannabis users (OR 4.1, 95% CI 1.3-13.4) |
| O'Sullivan, C. and O'Sullivan, C. (2004). "The psychosocial determinants of depression: a lifespan perspective." Journal of Nervous & Mental Disease 192(9): 585-594. | A cross-sectional study of 608 primary care patients aged over 18 years, from a rural community in Ireland (68% female) and a suburban community in Australia (69% female). | For females in Australia: currently depressed 35%; previously depressed 16%. Depression was measured using the CES-D. | The onset of depressive symptoms was positively associated with unfavourable childhood events, poorly perceived social supports, recent stressful life events, a vulnerable personality style, and previous depressive illness. |

1. ‘Oceanic ancestry groups’ was excluded from PsychInfo as it was not a recognised subject heading word and a key word search resulted in zero citations. [↑](#footnote-ref-1)
2. ‘ATSI’ or ‘CALD’ abbreviations were not used for the larger databases as they resulted in unrelated groups with the same acronym. [↑](#footnote-ref-2)
